# Enhanced dengue vaccine virus replication and neutralizing antibody responses in immune primed rhesus macaques

**DOI:** 10.1038/s41541-021-00339-y

**Published:** 2021-05-21

**Authors:** Michael K. McCracken, Caitlin H. Kuklis, Chandrika B. Kannadka, David A. Barvir, Mark A. Sanborn, Adam T. Waickman, Hayden C. Siegfried, Kaitlin A. Victor, Kristin L. Hatch, Rafael De La Barrera, Shannon D. Walls, Wiriya Rutvisuttinunt, Jeffrey R. Currier, Heather Friberg, Richard G. Jarman, Gregory D. Gromowski

**Affiliations:** 1 Viral Diseases Branch, Walter Reed Army Institute of Research, Silver Spring, MA USA; 2 Pilot Bioproduction Facility, Walter Reed Army Institute of Research, Silver Spring, MA USA

**Keywords:** Inactivated vaccines, Live attenuated vaccines, Dengue virus

## Abstract

Antibody-dependent enhancement (ADE) is suspected to influence dengue virus (DENV) infection, but the role ADE plays in vaccination strategies incorporating live attenuated virus components is less clear. Using a heterologous prime-boost strategy in rhesus macaques, we examine the effect of priming with DENV purified inactivated vaccines (PIVs) on a tetravalent live attenuated vaccine (LAV). Sera exhibited low-level neutralizing antibodies (NAb) post PIV priming, yet moderate to high in vitro ADE activity. Following LAV administration, the PIV primed groups exhibited DENV-2 LAV peak viremias up to 1,176-fold higher than the mock primed group, and peak viremia correlated with in vitro ADE. Furthermore, PIV primed groups had more balanced and higher DENV-1–4 NAb seroconversion and titers than the mock primed group following LAV administration. These results have implications for the development of effective DENV vaccine prime-boost strategies and for our understanding of the role played by ADE in modulating DENV replication.

## Introduction

Dengue virus (DENV) is a positive-sense RNA virus transmitted by mosquito vectors, primarily of the genus *Aedes*, consisting of four types in the genus *Flavivirus*. In humans, dengue is an acute febrile disease accompanied by a rash, headache, eye pain, and arthralgia, with laboratory findings of transient neutropenia, AST and ALT elevations and, less frequently, thrombocytopenia^1–5^. Approximately, 3.2 million cases of dengue fever were reported to the WHO in 2015, and unreported case estimates increase the annual global incidence to more than 50 million, including over 25,000 deaths due to more severe forms of the disease^6^. Secondary DENV infections are a well-known risk factor for severe dengue disease^7^. The presence of sub-neutralizing concentrations of antibodies that are cross-reactive between types, elicited by a primary DENV infection, is thought to result in enhanced viral replication after a second infection with a heterologous type. This phenomenon is termed antibody-dependent enhancement (ADE). The co-circulation of multiple DENV types in endemic regions and the potential for immune-mediated enhancement of viral replication necessitates a tetravalent vaccine, greatly complicating vaccine development efforts.

Three DENV live attenuated vaccines (LAV) are in advanced stages of development: TV003/TV005 from the U.S. National Institutes of Health, TAK-003 from Takeda, and Dengvaxia^TM^ from Sanofi Pasteur^8–11^. The TV003/TV005 vaccine consists of DENV-1, -3, and -4 components that are each attenuated by 3’ untranslated region deletion mutations engineered into recombinant viruses, while the DENV-2 component is based on a chimeric virus construct that incorporates the DENV-2 pre-membrane and envelope (prME) protein genes into the backbone of the DENV-4 vaccine virus^12–16^. The TAK-003 vaccine consists of a live attenuated DENV-2 component and 3 chimeric virus components constructed by incorporating the DENV-1, -3, and -4 prME protein genes into the recombinant DENV-2 vaccine backbone^17–20^. The NIH vaccine is in phase III clinical trials, and the Takeda vaccine has now completed phase III. Dengvaxia^TM^, a chimeric vaccine that expresses tetravalent DENV structural proteins in the yellow fever 17D virus construct, has the greatest global presence of the current dengue vaccine candidates and is the only dengue vaccine that has received U.S. FDA approval. The Sanofi vaccine was found to be approximately 60% efficacious overall in humans, and children who were seronegative to DENV when receiving the vaccine were found to be at greater risk for hospitalization and severe dengue disease from subsequent DENV natural infection compared to unvaccinated children^21–23^. The low efficacy of Dengvaxia^TM^ in DENV seronegative individuals, the possibility of more severe disease, and the current 3-dose vaccination schedule over the course of a year limit the utility of this vaccine for many travelers and U.S. military personnel^22,24,25^.

Tetravalent, live attenuated and inactivated vaccines have been pursued at the Walter Reed Army Institute of Research (WRAIR) in collaboration with industry partners^26^. The dengue purified inactivated vaccine (PIV) consists of DENV-1–4 virions purified and inactivated by formalin^27,28^. Phase I trials in the continental U.S. and Puerto Rico compared two doses of vaccine at months 0 and 1 with alum adjuvant or proprietary adjuvants, AS01E and AS03B^29,30^. The vaccine-elicited neutralizing antibody (NAb) responses to DENV-1–4 peaked at 4-weeks post dose two and waned thereafter. The DENV-1–4 LAV viruses developed at WRAIR were attenuated by serial passage in primary dog kidney (PDK) cells^31–43^. Phase I/II trials in the continental U.S., Puerto Rico, and Thailand compared various vaccine formulations at months 0 and 6. The vaccine elicited NAb responses to all four types, but typically with lower responses to DENV-1 and −3. A vaccination strategy wherein the tetravalent PIV is administered as the priming inoculation and the tetravalent LAV is subsequently administered as a boost in phase I trials was completed and published recently^44^. This PIV/LAV heterologous vaccine prime-boost approach led to higher seroconversion and more balanced DENV-1–4 NAb titers compared to vaccination with either PIV or LAV alone.

Importantly, the immune mechanisms behind the responses to the PIV/LAV approach are not yet known and raise the question of whether PIV priming that leads to effective LAV boosting might be achieved using a subset of the PIV viruses. A PIV/LAV prime-boost approach that requires only one or two PIV priming components could substantially reduce manufacturing costs. The goals of this study were to characterize antibody function post PIV priming, assess the impact of these antibodies on LAV replication, evaluate the quality of the immune responses post LAV boost, and, ultimately, determine the feasibility of reducing the number of PIV virus components required for this vaccination approach. DENV-1 and/or DENV-3 were chosen for the monovalent and bivalent formulations with the goal of improving the comparatively poor immune responses against these DENV types observed in clinical trials of the tetravalent LAV. Here, using a rhesus macaque model, we examined the effect of monovalent, bivalent, and tetravalent PIV priming on the replication of and immune responses to the tetravalent DENV LAV developed at WRAIR.

## Results

### Characterization of antibody elicited by PIV priming

**Fig. 1:**
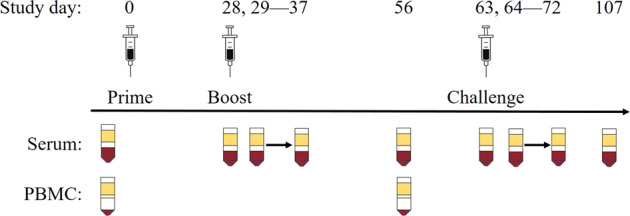
Vaccination schedule and serum collection by study day. Injections are indicated by a syringe. Serum and PBMC collections are indicated by their respective blood tube symbols.

Groups of 5 animals were immunized with tetravalent, bivalent (D1 + D3), or monovalent (D1 or D3) PIV, or were mock-immunized on study day 0. Blood was collected on study day 28, just prior to LAV booster immunization, in order to characterize antibody responses (Table 1 and Fig. 1).

**Table 1:** Group designation and immunization scheme by an animal.

Animal ID	Sex	Group	PIV immunization (day 0)	LAV immunization (day 28)	Challenge (day 63)
13U007	F	Tetra (D1–4) prime/LAV	Tetra (D1–4)	Tetra (D1–4)	DENV-2
12U021	M				
13U036	M				
13U063	M				
14U016	M				
13U043	F	Bi (D1 + D3) prime/LAV	Bi (D1 + D3)	Tetra (D1–4)	DENV-2
13U002	M				
13U040	M				
14U001	M				
14U019	M				
13U051	F	Mono (D3) prime/LAV	Mono (D3)	Tetra (D1–4)	DENV-2
13U015	M				
13U044	M				
14U002	M				
14U041	M				
13U052	F	Mono (D1) prime/LAV	Mono (D1)	Tetra (D1–4)	DENV-2
13U017	M				
13U048	M				
14U007	M				
14U049	M				
14U037	F	Mock prime/LAV	Mock	Tetra (D1–4)	DENV-2
13U019	M				
13U050	M				
14U011	M				
14U053	M				
14U043	F	Not immunized	None	None	DENV-2
13U024	M				
13U062	M				
14U012	M				
14U055	M

All of the PIV primed animals exhibited moderate to high levels of DENV-1–4 virion-reactive IgG antibody (Table 2 and Supplementary Table 1). In general, PIV primed groups exhibited moderately higher levels of IgG reactivity with DENV-2 and DENV-4, as compared to DENV-1 and DENV-3, regardless of the DENV type(s) used for priming. The monovalent (D3) primed group had 5.8-fold and 4.7-fold higher geometric mean titers (GMTs) to DENV-2 and DENV-4, respectively. The tetravalent PIV primed group had 4.2-fold higher GMTs for both DENV-2 and DENV-4. The bivalent primed group had 3-fold higher GMTs for both DENV-2 and DENV-4. The monovalent (D1) group had more balanced GMTs with a 3-fold higher GMT for DENV-2 and a 1.9-fold higher GMT for DENV-4. Unexpectedly, one animal in the mock primed group (13U019) exhibited a low level of IgG antibody reactivity to DENV-2 but not to the other DENV types. This animal did not have any detectable NAb to DENV-1–4, ZIKV, or WNV in the pre-study screening that was conducted, indicating that the low-level DENV-2 antibody reactivity was probably not due to prior exposure to this flavivirus.

**Table 2:** Endpoint anti-DENV IgG titers from day 28 post-PIV vaccination.

Group	Animal ID	DENV-1	DENV-2	DENV-3	DENV-4
Tetra (D1–4) prime	13U007	NT	7290	NT	7290
	12U021	270	2430	270	2430
	13U036	2430	7290	2430	7290
	13U063	2430	7290	2430	7290
	14U016	2430	7290	2430	7290
Bi (D1 + D3) prime	13U043	2430	7290	2430	7290
	13U002	2430	7290	2430	7290
	13U040	810	2430	810	2430
	14U001	2430	7290	2430	7290
	14U019	2430	7290	2430	7290
Mono (D3) prime	13U051	270	2430	270	810
	13U015	270	810	270	810
	13U044	810	7290	810	7290
	14U002	810	2430	810	2430
	14U041	270	2430	270	2430
Mono (D1) prime	13U052	2430	7290	2430	810
	13U017	810	2430	810	2430
	13U048	810	7290	810	7290
	14U007	2430	7290	2430	7290
	14U049	2430	2430	2430	2430
Mock prime	14U037	<30	<30	<30	<30
	13U019	<30	810	<30	<30
	13U050	<30	<30	<30	<30
	14U011	<30	<30	<30	<30
	14U053	<30	<30	<30	<30

Reciprocal dilutions are shown. The lowest dilution tested was 1/30. NT = sample not tested due to volume limitations.

**Fig. 2:**
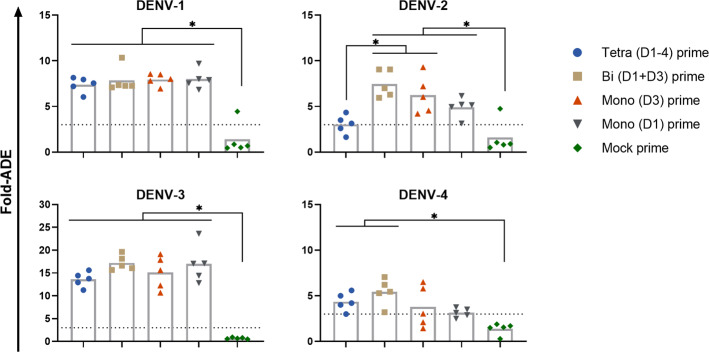
In vitro enhancement of DENV infection by sera from day 28 post-PIV vaccination. Each of the four DENV types was mixed with a 1/8 dilution of sera from each group, and the infectivity of each was measured relative to the virus alone (fold-ADE). Bar heights are the means for each group. Data were analyzed using a one-way ANOVA model with Tukey’s multiple comparisons test. *p < 0.05.

An in vitro ADE assay with BHK cells that express human CD32a was used to measure infection enhancing antibodies at biologically relevant, low serum dilutions; importantly, the recognition of rhesus IgG was found previously to be very similar between human and rhesus CD32a variants^45^. The PIV primed animals exhibited similar, relatively high in vitro ADE activity across groups for DENV-1 (7.8 mean fold-ADE) and DENV-3 (15.7 mean fold-ADE). More variable levels of in vitro ADE for DENV-2 were observed across PIV primed groups, with the tetravalent primed group (3.5 mean fold-ADE) being significantly lower compared to the bivalent and monovalent (D3) primed groups (7.5 and 6.3 mean fold-ADE, respectively). Lower levels of in vitro ADE were observed for DENV-4, with the tetravalent (4.4 mean fold-ADE) and bivalent (5.4 mean fold-ADE) groups being the highest (Fig. 2 and Supplementary Fig. 1). Serum from animal 13U019 in the mock primed group also exhibited ADE activity to DENV-2 and to a lesser extent DENV-1 at relatively low serum dilutions, which is consistent with the low-level IgG reactivity to DENV-2 observed by ELISA.

**Fig. 3:**
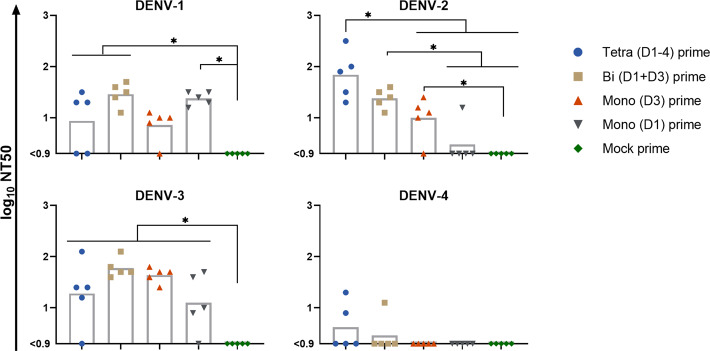
50% neutralization titers against DENV-1–4 of sera from day 28 post-PIV vaccination. Log_10_-transformed data are shown. The lowest dilution tested was 1/8. Bar heights are the geometric means for each group. Data were analyzed using a one-way ANOVA model with Tukey’s multiple comparisons test. *p < 0.05.

A focus-reduction neutralization test (FRNT) assay was used to measure NAb at low serum dilutions (Fig. 3). All of the PIV primed animals had NAb to at least one DENV type, with the greatest number of animals having NAb to DENV-1 (17 of 20), DENV-2 (15 of 20), and DENV-3 (18 of 20), and the least having NAb to DENV-4 (3 of 20). In general, the NT50 titers were relatively low (<100) for animals receiving all PIV priming regimens. Against DENV-1 and DENV-3, none of the primed groups differed significantly from each other. Against DENV-2, the NAb GMT for the tetravalent primed group was significantly higher than that of the monovalent (D3) and monovalent (D1) primed groups and was statistically similar to the bivalent primed group; the bivalent and monovalent (D3) primed groups were also significantly higher than the mock primed group. Meaningful comparisons could not be made for DENV-4. There was a disproportionately large quantity of binding IgG antibody compared to neutralizing antibody against DENV-2 and DENV-4 in most of the primed groups, with mean ratios of between 293 and 2544/1 (Supplementary Fig. 2). In contrast, the ratio of binding IgG to neutralizing antibody against DENV-1 and DENV-3 was considerably smaller, with mean ratios ranging from 68 to 115/1 against DENV-1, and from 11 to 88/1 against DENV-3. The two exceptions were the tetravalent primed group against DENV-2, which had a smaller mean ratio of 127/1, and the monovalent (D1) primed group against DENV-3, which had a larger mean ratio of 182/1. The mean ratio for the monovalent (D3) primed group against DENV-3, which was 11/1 and the lowest of any primed group and DENV type combination, is particularly interesting because it indicates that a substantial portion of the binding antibody, 9.1%, was neutralizing, and yet the in vitro fold-ADE of all primed groups was highest against DENV-3. In contrast, the other homologous combination of monovalent (D1) prime against DENV-1 had a mean ratio of 80/1, indicating that only 1.25% of the binding antibody was neutralizing.

### LAV virus replication

**Fig. 4:**
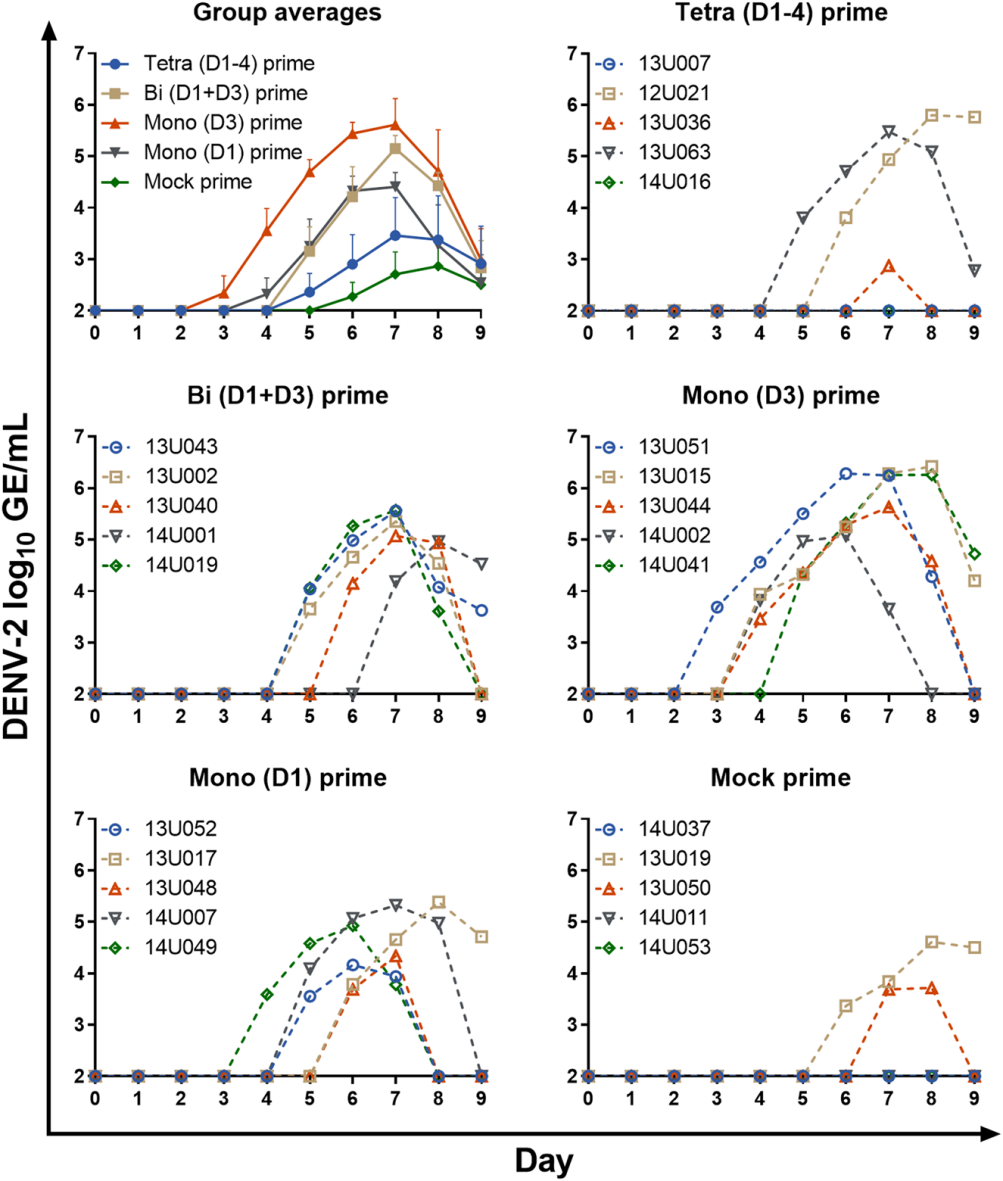
Viremia of DENV-2 live-attenuated vaccine virus. Sera from days 0–9 post-LAV boost were tested by qRT-PCR for replication of the DENV-1–4 vaccine viruses. Only replication of DENV-2 was detectable. Data are presented as log_10_-transformed genome equivalents (GE)/mL. Both group geometric means and data for individual animals within each group are shown. Data were analyzed using a mixed-effects model with Geisser–Greenhouse correction and Dunnett’s multiple comparisons test. Error bars show the standard error of the means.

**Fig. 5:**
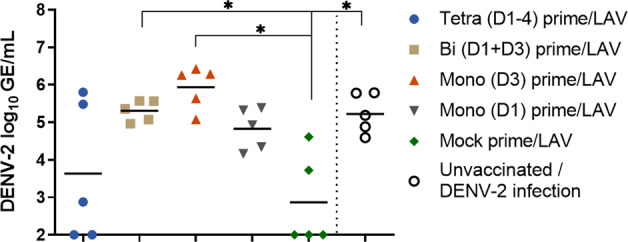
Peak viremia for DENV-2 live-attenuated vaccine virus and wild-type challenge virus. Shown are the peak viremia values from each animal following LAV vaccination or the following wild-type DENV-2 infection of unvaccinated control animals. Data are presented as log_10_-transformed genome equivalents (GE)/mL. Horizontal bars are the geometric means for each group. Data were analyzed by Welch’s ANOVA model and Dunnett’s T3 multiple comparisons test. *p < 0.05.

The PIV primed and mock primed groups of animals were all immunized on day 28 with LAV. Vaccine virus replication in the sera was determined by quantitative reverse transcription-polymerase chain reaction (qRT-PCR) for 9 days post-LAV administration. Only the DENV-2 vaccine virus produced detectable viremia in any of the animals (Fig. 4). The tetravalent PIV primed group and the mock primed group had 3/5 and 2/5 animals with detectable DENV-2 LAV viremia, respectively. The bivalent and both monovalent PIV primed groups had 5/5 animals with detectable DENV-2 LAV viremia. Any visually apparent differences in geometric mean viremia between the tetravalent PIV primed group as compared to the mock primed group were not significantly different, likely due to the relatively low number of animals with detectable replication. In contrast, as compared to the mock primed group, the geometric mean viremia was significantly greater in the monovalent (D3) primed group on days 4–7, in the monovalent (D1) primed group on days 6–7, and in the bivalent primed group on day 7. The peak viremia value from each animal was also compared among groups (Fig. 5). The geometric means of peak viremias were significantly greater in the bivalent primed (275-fold) and monovalent (D3) primed (1178-fold) groups as compared to the mock primed group. The geometric means of peak viremias were also greater for the monovalent (D1) (92-fold) and tetravalent (5.8-fold) primed groups as compared to the mock primed group but were not significantly different. Interestingly, in the tetravalent primed group, there was a stark contrast in peak viremia for 2/5 animals that had very high peak viremia and 3/5 animals had low/undetectable viremia. The other PIV primed groups had more consistent, high peak viremias by comparison.

### Correlations between in vitro ADE and viremia

In order to explore the potential relationship between in vitro ADE and viremia, a series of correlations and regression analyses on various parameters associated with these two datasets were performed, irrespective of group. The fold-increase in infectivity (fold-ADE) measured in vitro for each day 28 serum sample at a 1/8 dilution was used as the independent variable, and the Spearman correlations between these values and the animals’ corresponding log_10_-transformed peak viremia values and durations of viremia (days) were determined. A significant correlation was found for both of these combinations, with a Spearman *r* = 0.6898 (*p* = 0.0002) for the peak viremia and a Spearman *r* = 0.6126 (*p* = 0.0011) for the duration of viremia.

**Fig. 6:**
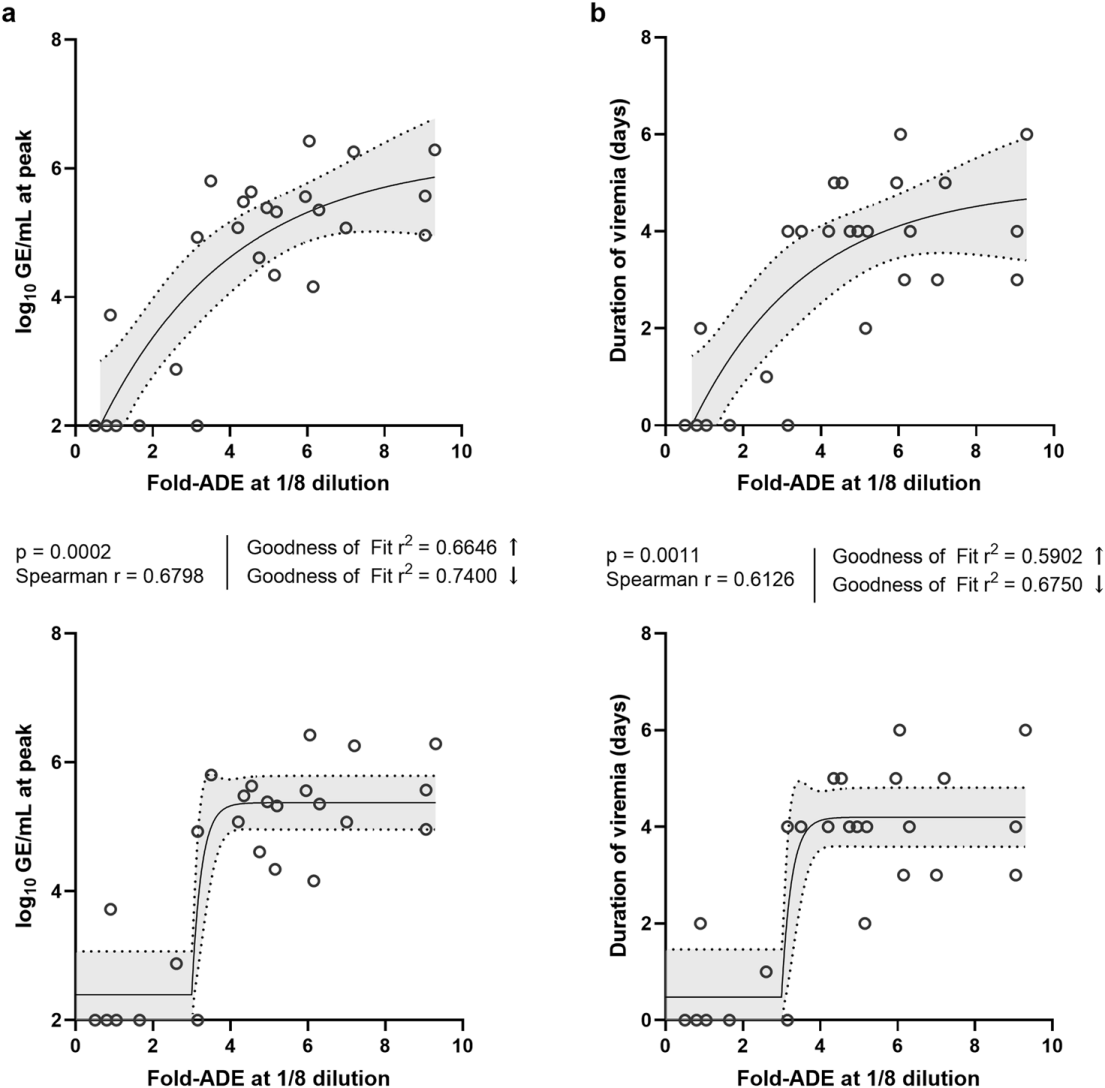
Relationships between in vitro ADE and viremia. Spearman correlations and non-linear regression analyses between in vitro fold-ADE of day 28 sera at a 1/8 dilution and either peak LAV viremia (**a**) or duration of LAV viremia (**b**) were performed. The upper panels show the data fit with an exponential, one-phase association curve with unconstrained parameters. The lower panels show the data fit similarly, but using an initial plateau region constrained to a fold-ADE value of 3. Use of the initial plateau region increased the Goodness of Fit *r*^2^ value by >11%. Shaded regions represent 95% confidence intervals.

Curves were then fit to the correlated data by nonlinear regression using two different approaches. The fold-ADE vs. peak viremia data was first to fit using an exponential, one-phase association with unconstrained parameters, which fit the data reasonably well visually and yielded an *r*^2^ = 0.6646 (Fig. 6A). The data were then fit again using an exponential curve, but this time utilizing a plateau region followed by one-phase association; the right side of the plateau was constrained to a fold-ADE value of 3, which is the maximum value measured against any DENV type in the in vitro ADE assay for a negative serum sample. This second method increased the fit to *r*^2^ = 0.7400. These same two approaches were also applied to the fold-ADE vs. duration of viremia data, yielding *r*^2^ = 0.5902 and *r*^2^ = 0.6750, respectively (Fig. 6B).

### The NAb response to different heterologous vaccine prime-boost regimens

**Fig. 7:**
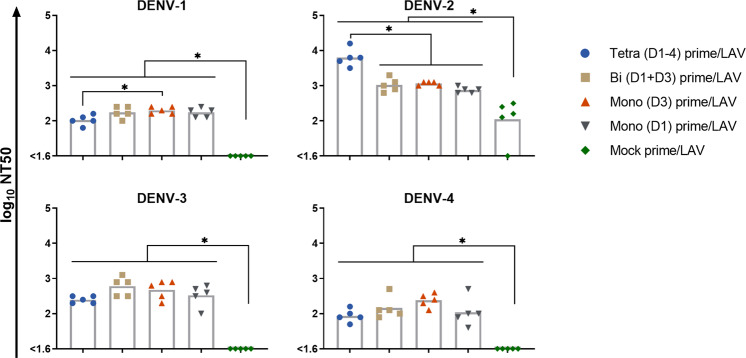
Neutralizing antibody response following LAV vaccination. Blood samples collected on day 28 post LAV vaccination (study day 56) were assessed for the neutralizing antibody titer of each group against each DENV type. Neutralizing antibody titers are presented as log_10_-transformed NT50. The lowest dilution tested was 1/40. Bar heights are the geometric means for each group. Data were analyzed using a one-way ANOVA model with Tukey’s multiple comparisons test. *p < 0.05.

The DENV-1–4 NAb GMTs and seroconversion rates were determined for day 56, which is 28 days after immunization with LAV (Fig. 7). The mock primed group had a modest NAb GMT of 110 for DENV-2 and only 4 of 5 animals seroconverted to this type. There was no NAb seroconversion to DENV-1, -3, and -4 in this group. By comparison, the PIV primed groups all had significantly higher NAb GMTs for DENV-1–4 and there was complete tetravalent seroconversion. The GMTs were similar across PIV primed groups for each DENV type, with two exceptions: a significantly lower NAb GMT for the tetravalent primed group compared to the monovalent (D3) primed group for DENV-1, and a significantly higher NAb GMT for the tetravalent primed group compared to the other PIV primed groups for DENV-2. The NAb GMTs for PIV primed groups compared to the mock primed group ranged from >3-fold to >5-fold higher for DENV-1, 7-fold to 58-fold higher for DENV-2, >8-fold to >15-fold higher for DENV-3, and >2-fold to >6-fold higher for DENV-4. The NAb GMTs for all groups were highest for DENV-2, which was the dominant replicating vaccine virus. The tetravalent primed group was particularly biased towards DENV-2 NAb with GMTs for the other types being 13-fold to 72-fold lower. The other PIV primed groups had more balanced NAb GMTs against DENV-1, -3, and -4 which were only 2-fold to 7-fold lower than the DENV-2 NAb GMTs.

### Type-specific NAb responses

**Fig. 8:**
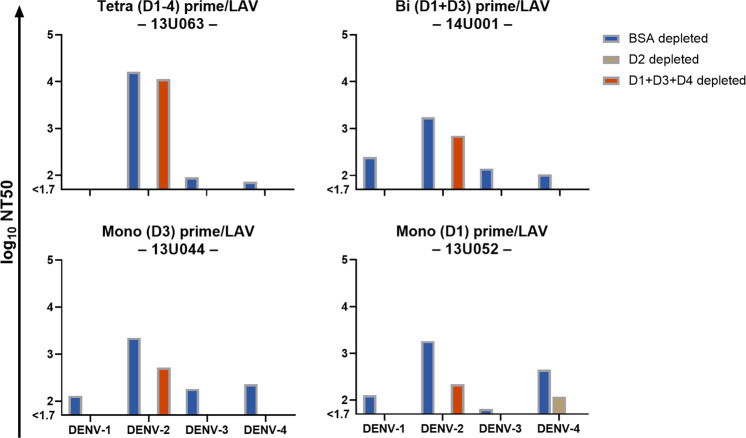
Type-specific neutralizing antibody responses of representative animals in each PIV primed/LAV boosted group. Sera were depleted of antibody by sequential rounds of incubation with polystyrene magnetic particles coated with BSA (control depleted), DENV-2 virions (D2 depleted), or DENV-1, -3, and -4 virions (D1 + D3 + D4 depleted). Neutralizing activity of depleted sera was tested by FlowNT. Data are presented as log_10_-transformed NT50 values against each DENV type.

We hypothesized that the observed dominant DENV-2 vaccine virus replication would primarily elicit type-specific NAb responses to DENV-2 and cross-reactive responses to the other DENV types in PIV primed animals. Therefore, we performed serum antibody depletions with D2-only, D1 + D3 + D4 combination, or bovine serum albumin (BSA)–control on a representative animal from each vaccinated group (Fig. 8). The relative levels of type-specific and cross-reactive NAb were determined based on a reduction in NAb titer following heterologous DENV antigen depletion. Variability of up to threefold between assays is common for neutralization assays. Accordingly, a reduction of ≤3-fold compared to control depleted sera was interpreted as predominantly type-specific NAb, >3-fold reduction interpreted as a mixture of type-specific and cross-reactive NAb, and a complete reduction (titer < 40) interpreted as predominantly cross-reactive NAb, in accordance with the previous analysis^46^; the results of these interpretations are shown in Table 3. All four animals exhibited cross-reactive NAb against DENV-1 and DENV-3 (with the exception of 13U063, which was below the limit of detection for DENV-1). Animals 13U063 and 14U001, of the tetravalent prime and bivalent prime groups, respectively, exhibited primarily type-specific NAb against DENV-2, whereas the animals of the monovalent prime groups (13U044 and 13U052) exhibited a mixture of both cross-reactive and type-specific NAb against DENV-2. Only animal 13U052 of the monovalent (D1) prime/LAV boost group exhibited detectable type-specific NAb to a different DENV type as well, with a mixture of both cross-reactive and type-specific NAb against DENV-4.

**Table 3:** Predominant neutralizing antibody specificity against each DENV type for a representative animal in each PIV primed/LAV boosted group.

	DENV-1 Ab	DENV-2 Ab	DENV-3 Ab	DENV-4 Ab
**13U063** **(Tetra D1–4)**	n.d.	Type-specific	Cross-reactive	Cross-reactive
**14U001** **(Bi D1** + **D3)**	Cross-reactive	Type-specific	Cross-reactive	Cross-reactive
**13U044** **(Mono D3)**	Cross-reactive	Mixture	Cross-reactive	Cross-reactive
**13U052** **(Mono D1)**	Cross-reactive	Mixture	Cross-reactive	Mixture

*n.d.* not detected

### ELISpot against DENV virions and rE proteins

DENV-specific memory B cell responses were also determined at Day 56 post-vaccination (28 days after immunization with LAV). All PIV primed groups showed responses to all four DENV types as assessed by binding to whole virions and/or recombinant E proteins. There were no remarkable differences among groups. No mock primed animals demonstrated detectable DENV-specific memory B cell responses (Supplementary Fig. 3).

### DENV-2 challenge

**Fig. 9:**
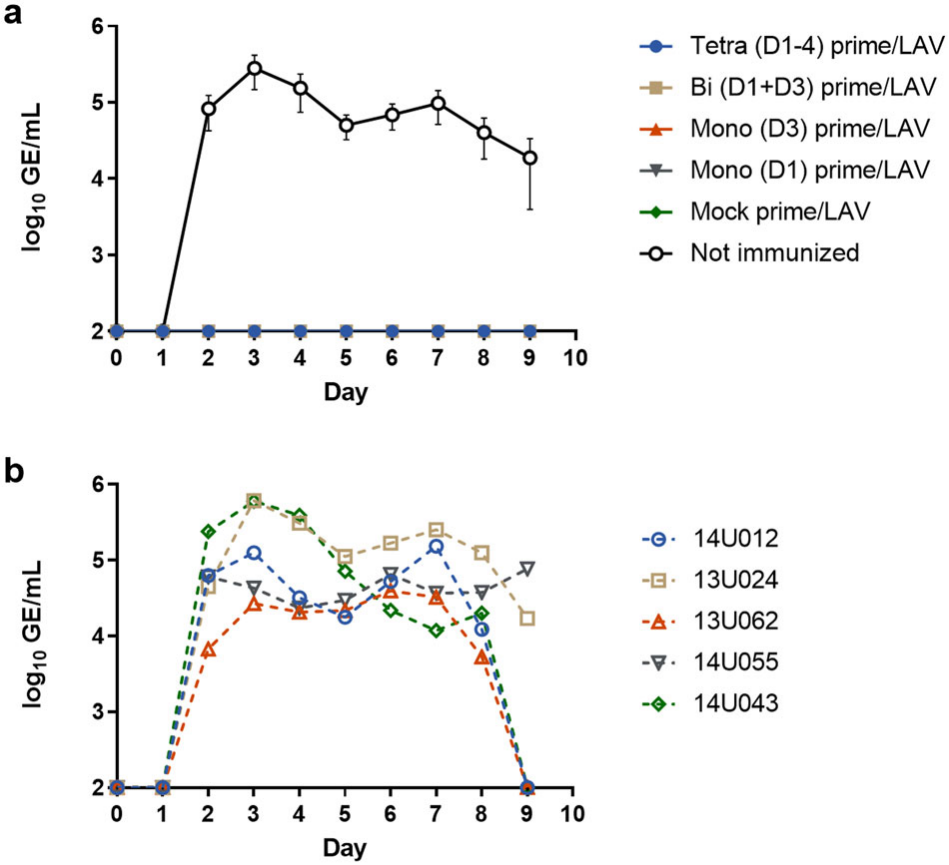
Viremia resulting from the challenge of vaccinated and unvaccinated control animals with wild-type DENV-2. All vaccinated animals were protected from detectable viremia upon challenge. All unvaccinated control animals had detectable DENV-2 viremia lasting from day 2 until day 8 or beyond. Data are presented as log_10_-transformed genome-equivalents (GE)/mL. **a** Geometric mean viremia by the group; error bars show the standard error of the means. **b** Viremia curve of each animal in the unvaccinated control group shown in panel (**a**).

As the mock primed, LAV boosted group only had detectable NAb to DENV-2, we chose to challenge all groups with this DENV type in order to determine how vaccine-induced immunity translated to protection from challenge. All groups of animals were challenged with a non-attenuated DENV-2 strain on study day 63, which was 35 days post LAV boost. None of the vaccinated animals had detectable challenge virus viremia, as measured by qRT-PCR, whereas all 5 unvaccinated control animals had detectable viremia (Fig. 9).

The post-challenge NAb titers were also determined in order to identify breakthrough challenge virus replication that was not detected by the qRT-PCR assay (Table 4). Of all the vaccinated animals in all groups, only animal 14U011 in the mock primed group exhibited a rise in NAb titer post-challenge. This animal had a pre-challenge NAb titer of <40 and a post-challenge NAb titer of 328, which is a >8.2-fold rise and may be indicative of challenge virus replication. None of the other vaccinated animals had a meaningful change in NAb titer and were therefore protected from the DENV-2 challenge. Taken together, the undetectable viremia and lack of NAb boost from the DENV-2 challenge demonstrated the non-inferiority of the monovalent and bivalent PIV priming regimens compared to the tetravalent PIV priming.

**Table 4:** Neutralizing antibody titers pre- and post-challenge with wild-type DENV-2.

Group	Animal ID	Pre-challenge NT50^a^	Post-challenge NT50^b^	NT50 fold-change
Tetra (D1–4) prime/LAV	13U007	4476	541	0.1
	12U021	6143	1259	0.2
	13U036	3323	957	0.3
	13U063	6116	2029	0.3
	14U016	14,789	2764	0.2
Bi (D1 + D3) prime/LAV	13U043	907	1159	1.3
	13U002	779	326	0.4
	13U040	619	244	0.4
	14U001	1955	426	0.2
	14U019	1321	572	0.4
Mono (D3) prime/LAV	13U051	1136	644	0.6
	13U015	1311	587	0.4
	13U044	1015	595	0.6
	14U002	1111	729	0.7
	14U041	1406	619	0.4
Mono (D1) prime/LAV	13U052	816	266	0.3
	13U017	715	296	0.4
	13U048	981	316	0.3
	14U007	636	568	0.9
	14U049	575	489	0.9
Mock prime/LAV	14U037	149	109	0.7
	13U019	305	158	0.5
	13U050	124	205	1.7
	14U011	<40	328	>8.2
	14U053	255	174	0.7
Not immunized	14U043	<40	814	>20.3
	13U024	<40	2122	>53.1
	13U062	<40	518	>13.0
	14U012	<40	621	>15.5
	14U055	<40	419	>10.5

Titers are presented as NT50. Fold-change values between pre- and post-challenge are presented. The lowest dilution tested was 1/40.

^a^Sera were collected 28 days post LAV immunization.

^b^Sera were collected 43 days post-DENV-2 challenge.

We also compared the DENV-2 challenge virus viremia in unvaccinated control animals to the DENV-2 LAV boost viremias in all groups. Importantly, the DENV-2 challenge strain is the non-attenuated parent of the DENV-2 LAV strain. The DENV-2 challenge strain geometric mean viremia peak occurred 4–5 days earlier than the DENV-2 LAV. Also, in the mock primed group, the DENV-2 LAV had a significantly lower geometric mean viremia peak compared to the DENV-2 non-attenuated parent strain, in accordance with its attenuated phenotype. However, the DENV-2 LAV geometric mean viremia peak observed for the bivalent and both monovalent PIV primed groups were similar to or higher than that of the DENV-2 non-attenuated parent strain in the unvaccinated control animals. Taken together with the antibody data presented above, this indicates that infection-enhancing antibody caused the DENV-2 LAV to reach or exceed viremia levels achieved by the non-attenuated parent strain (refer to Fig. 5).

## Discussion

In this study, we used a rhesus macaque model to examine the effect of monovalent, bivalent, and tetravalent PIV priming on the replication of and immune responses to a DENV-1–4 tetravalent LAV. Animals primed with bivalent or either of the monovalent formulations of PIV produced a level of DENV-2 LAV viremia peak, as well as NAb titers, that were comparable to that of the DENV-2 challenge virus in unvaccinated control animals. Despite the demonstration of in vitro ADE for all four DENV types, ADE only appeared to influence in vivo replication of the DENV-2 vaccine virus, which was the only vaccine virus that detectably replicated in the rhesus macaques. Our results corroborate previous observations that the DENV-1 and -3 vaccine viruses are unable to replicate to detectable levels in rhesus macaques, and therefore could not benefit in vivo from the additional target cell attachment and entry options bestowed by infection enhancing antibody detected in vitro^47^. There was evidence that the DENV-4 LAV virus replicated in a single animal in the monovalent (D1) primed group, as it developed antibodies specific to this type. This is in contrast to the replication of these vaccine viruses in human clinical trials, where DENV-4 is the predominant type, followed by DENV-2, and DENV-1 and -3 were only detected in two and one subjects, respectively, across all trials^40–44^. Overall, in vivo replicative fitness of the DENV strain coupled with the presence of infection enhancing antibody was a potent combination of factors that increased levels and duration of viremia in the present study.

Other groups have also demonstrated DENV ADE in the rhesus macaque model. A prior vaccination study with this PIV followed by a non-attenuated DENV challenge demonstrated enhancement of peak viremia in some groups for DENV-1 that were approximately 10-fold higher than the placebo group; no increase in viremia was observed for DENV-2 challenge in that study^28^. Another PIV vaccination study demonstrated enhancement of both non-attenuated DENV-1 and DENV-2 peak viremia in vaccinated groups by as much as approximately sevenfold over unvaccinated groups^48^. ADE of DENV replication has also been demonstrated in rhesus macaques resulting from sequential infection, passive transfer of DENV-2-immune human cord-blood serum, and administration of an anti-DENV cross-reactive monoclonal antibody^49–51^. The highest increase in viremia titers from these prior studies was approximately 100-fold, with DENV-4. In our study, monovalent (D1 or D3) PIV priming increased DENV-2 LAV viremia by approximately 90–1200-fold that of the unprimed group of animals. This observation underscores the biological significance of heterologous DENV exposures that can elicit poorly neutralizing, cross-reactive DENV antibodies that are potent mediators of ADE.

Correlation and regression analyses of in vitro ADE and viremia data suggest that the presence of detectable infection-enhancing antibodies in a low dilution of serum may be predictive of viremia peak and duration for the DENV-2 LAV. More importantly, it appeared that the relative magnitude of in vitro enhancement was less impactful than the presence or absence of infection enhancement above a threshold. Based on these data, we hypothesize that the presence of any meaningful level of infection enhancement gives the virus—DENV-2 in this case—sufficient access to target cells that bear Fc-gamma receptor IIa (CD32a). This would substantially increase the number of infected cells in blood and lymph, resulting in a uniform increase in viremia peak and duration, as observed in this study. This observation also has implications for natural infection and risk of more severe disease in humans.

Heterologous prime-boost strategies have been in development for some years now against many viral pathogens, including DENV and Japanese encephalitis virus (JEV)^52–56^. These strategies include the use of inactivated viruses, protein or subunit antigens, DNA that codes for a viral protein, chimeric viruses, and attenuated viruses in various combinations and schedules, and often produce greater and/or more balanced immune responses as compared to vaccination with one component alone or a homologous prime-boost regimen. These heterologous prime-boost strategies have been reviewed extensively^57–59^, and include cross-species immunization regimens as in the case of JEV or yellow fever virus followed by DENV or tick-borne encephalitis virus^60–62^.

A heterologous DENV vaccine prime-boost regimen like the one tested in this study has clear advantages that include better vaccine virus replication (in the case of DENV-2) and higher NAb titers. Indeed, the recently published clinical trial showed that tetravalent PIV prime followed by tetravalent LAV boost resulted in 100% seroconversion to each DENV type by day 28 post-LAV, with balanced NAb GMT^44^. In contrast, across all prior clinical trials of this tetravalent LAV product, a single dose of LAV produced seroconversion rates ranging from 28% for DENV-1 to 63% for DENV-2, and unbalanced GMT favoring DENV-4^36,38–43^. A limitation in the present study was that the DENV-1, -3, and -4 vaccine viruses did not replicate detectably, even in animals with infection-enhancing antibodies. This resulted in moderate to low level cross-reactive NAb that are unlikely to provide long-term protection^63^. However, if a PIV prime is paired with an LAV containing all four DENV types that can consistently and productively infect humans, this approach could result in a substantially more immunogenic vaccine. A potential risk is that ADE of LAV viruses could increase reactogenicity. However, DENV LAVs run the same risk during vaccination campaigns in DENV endemic areas where prior immunity is common. A critical advantage of vaccinating immunologically naïve individuals with a PIV/LAV regimen is that higher titer cross-reactive and type-specific NAbs might be elicited, which is more analogous to natural sequential exposures, and this could be accomplished in a relatively short 0, 1-month immunization schedule^63,64^. This approach could be particularly effective for travelers and military personnel that do not reside in DENV-endemic areas. Importantly, bivalent and monovalent PIV priming appeared to be effective at eliciting DENV-1–4 NAb post LAV boost, thus tetravalent priming might not be required to get optimal, balanced responses.

Overall, our results have implications for the development of effective DENV vaccine prime-boost strategies and contribute to our understanding of the role played by ADE in modulating DENV replication in the rhesus macaque model. Future studies will focus on the role of ADE as well as other properties of DENV antibodies in human volunteers immunized with the tetravalent PIV/LAV prime-boost regimen in previously completed clinical studies^44^.

## Methods

### Ethics Statement

The research was conducted under an animal use protocol approved by the WRAIR/NMRC Institutional Animal Care and Use Committee in an AAALACi accredited facility, in compliance with the Animal Welfare Act and other federal statutes and regulations relating to animals and experiments involving animals, and adheres to principles stated in the *Guide for the Care and Use of Laboratory Animals*, NRC Publication, 2011 edition.

Animals were pair-housed on a 12:12-h light cycle and with 10–15 room air changes per hour. Animals were fed Old World Primate Chow 5038 (Quality Lab Products, Elkridge, MD) twice daily, fresh fruit at least three times a week, and water *ad libitum*. Environmental Enrichment was provided in the form of cage complexities, food treats, opportunities to forage, a rotation of several toys and puzzles, periodic access to an activity cage that permits climbing, jumping, and swinging, and alternating days of television and music. Animals were anesthetized by intramuscular injection with a combination of ketamine (5 mg/kg) and dexmedetomidine (0.01 mg/kg) prior to all procedures, and anesthesia was reversed by intramuscular injection of atipamezole (0.01 mg/kg). The studies performed under this protocol were nonterminal.

### Animals

Acquisition of healthy, adult, male and female, Indian origin, colony bred rhesus macaques were obtained from Covance Research Products (Alice, TX). Prior to being placed on protocol all animals were tested and found negative for neutralizing antibodies to WNV, ZIKV, and DENV-1–4. Animals were assigned in no particular order, while attempting to balance ages and weights, to six experimental infection groups outlined in Table 1. The vaccination schedule for each group is shown in Fig. 1. Blood samples were collected before and after each vaccination and/or DENV-2 challenge. The vaccine prime was given intramuscularly in the upper arm, while the vaccine boost and DENV-2 challenge were administered subcutaneously in the upper arm. Weight, body temperature, pulse, and respiration rates were monitored for each animal at every time point.

### Vaccines

The dengue PIV consist of purified, formalin-inactivated DENV-1 WestPac-74, DENV-2 S16803, DENV-3 CH53489, and/or DENV-4 TVP/360 virions^27,28^. The LAV viruses—DENV-1 45AZ5, DENV-2 S16803, DENV-3 CH53489, DENV-4 341750—were attenuated by serial passage in PDK cells^31–43^. Replication capacity of DENV-1–4 viruses in the LAV vaccine was confirmed in Vero (African green monkey kidney) and C6/36 (*Aedes albopictus* mosquito) cell cultures prior to study start (Supplementary Table 2).

### Viruses and cells

The virus strain used for in vivo challenge was DENV-2 S16803. The virus strains used for in vitro assays were DENV-1 West Pac 74 (GenBank accession U88535), DENV-2 S16803 (GenBank accession GU289914), DENV-3 CH53489 (GenBank accession DQ863638), DENV-4 TVP-360 (GenBank accession KU513442), ZIKV Paraiba-01 (GenBank accession KX280026), and rWN/DEN4Δ30 (see ref. ^65^). The DENV strains match the strains used to formulate the PIV vaccines. Infectious virus stocks were produced by passage in *Aedes albopictus* mosquito C6/36 cells as described previously^66^. Purified virus stocks for use as in vitro antigens were amplified in Vero cells and purified by ultracentrifugation over a 30% sucrose solution as described previously^66^. Vero cells were grown in Eagle’s Minimum Essential Medium containing 10% fetal bovine serum (FBS), 1% Penicillin–Streptomycin, 1% l-glutamine, and 1% sodium bicarbonate at 37 °C with 5% CO_2_. C6/36 cells were grown in M199 containing 10% FBS and 1% Penicillin–Streptomycin at 30 °C with 5% CO_2_. BHK-21 cells (ATCC CCL-10) were obtained from ATCC and transfected using Lipofectamine 2000 (ThermoFisher Scientific) with a CD32a expression vector (CloneID OHu27189D, GenScript). Fluorescence-activated cell sorting was used to sort single CD32a-expressing cells, detected using an anti-human CD32a mAb (Clone FLI8.26, BD Biosciences), into single wells of a 96-well plate. Clones were allowed to expand and then screened by flow cytometry for CD32a high-expressers (Supplementary Fig. 4). A single high-expressing clone, hereafter called BHK(CD32a), was selected, expanded by multiple passages, and used for in vitro ADE assays described below. BHK (CD32a) cells were maintained in Dulbecco’s Modified Eagle Medium containing 5% FBS, 1% Penicillin–Streptomycin, 1% l-glutamine, 1% NEAA, and 1% Geneticin at 37 °C with 5% CO_2_. BHK (CD32a) cells are available from the corresponding author on reasonable request.

### ELISA

The binding of immune sera to purified DENV-1–4 was measured as described previously^67^, starting at a 1/30 dilution. The detection antibody was goat anti-human IgG (γ-chain specific) conjugated to peroxidase (Sigma A6029). End-point titers were determined as the reciprocal of the final dilution at which the optical density (OD) was greater than 1.5× background. The background was determined by calculating the mean of the ODs of sera from four naïve animals at each dilution.

### Neutralization assays

Standard plaque-reduction neutralization tests on Vero cell monolayers were used to screen pre-study sera for determining serologically WNV-naïve animals at a 1/10 dilution of heat-inactivated sera.

FRNT on Vero cell monolayers in 96-well plates were used to determine titers in heat-inactivated sera, following PIV vaccination, in order to use lower dilutions of sera; previously described by Priyamvada et al.^68^. Data were analyzed by nonlinear regression to determine 50% neutralization titers in GraphPad Prism 8.

Neutralizing antibody titers in heat-inactivated sera, following LAV vaccination and DENV-2 challenge, were determined using a flow cytometry-based neutralization (FlowNT) assay in U937 cells expressing DC-SIGN as previously described^69,70^. This assay was also used to screen pre-study sera for determining ZIKV and DENV-1–4 serologically naïve animals. Data were analyzed by nonlinear regression to determine 50% neutralization titers in GraphPad Prism 8.

### ADE assay

In vitro, ADE of DENV infection was quantified in BHK cells expressing human CD32a. Beginning at neat, twofold serial dilutions of heat-inactivated sera were incubated with a virus (in sufficient amount to infect approximately 1% of BHK [CD32a] cells) at 1:1 for 1 h at 37 °C. Duplicate wells of a 96-well plate containing confluent BHK (CD32a) cells were then infected with 30 µL of this mixture. Cells were infected and incubated for 48 h in a 37 °C, 5% CO_2_, humidified incubator. The medium was removed, a single cell suspension was prepared, and then processing, immunostaining, and quantification continued as referenced above for the FlowNT assay. Fold-infection relative to control serum is reported (fold-ADE). A maximum fold-ADE of 3 was observed for any negative control serum sample tested for any DENV type; accordingly, a fold-ADE value of 3 was used as the cutoff between positive and negative samples. The mean fold-ADE measured in vitro for each serum sample at the lowest dilution that could be reliably assayed, 1/8, was used for comparisons between groups.

### Viremia determination by qRT-PCR

The quantity of DENV in sera was determined as previously described, with modification: the DENV-2 probe fluorophore was changed to JOE^71^. RNA was extracted from sera using the MagMAX 96 Viral RNA Kit, and RNA was extracted from cell culture media using the MagMAX Pathogen RNA/DNA kit, on the KingFisher Flex Purification System. Eluted RNA was quantified by qRT-PCR using the SuperScript III Platinum One-Step qRT-PCR kit (Thermo Fisher Scientific) on the QuantStudio 7-Flex Real-Time PCR instrument (Thermo Fisher Scientific). DENV RNAemia was calculated as genome equivalents (GE) per mL using an internal standard curve of 10-fold serially diluted in vitro-transcribed RNA. The limit of quantitation, defined here as detection of a standard curve dilution in ≥95% of at least 20 replicates curves tested, for the DENV-2 assay is 50 GE/reaction. Values for undetectable sera samples were replaced with 100 GE/mL for the purposes of statistical analysis and graphing; this value is one-half of a theoretical minimum of one detected copy per reaction multiplied by the assay dilution factor of 200 to achieve per mL concentrations.

### Depletion of DENV-specific antibodies from immune sera

The antibody depletion method was carried out as described previously^70^, with modification. Purified DENV or BSA control protein in 1× phosphate-buffered saline (PBS), pH 7.4 was adsorbed onto 3.0–6.0 µm SPHERO polystyrene cross-linked magnetic particles by overnight incubation at 4 °C. The control and DENV-adsorbed beads were blocked with BSA (10 mg/mL) in PBS for 2 h at room temperature, with continuous inversions, and washed twice with PBS. Immune sera were depleted of DENV-specific antibody by incubation with virus-adsorbed beads for 2 h at room temperature, with continuous inversions. Sequential rounds of antibody depletion were performed, and the sera were tested for successful removal of the desired DENV-specific antibodies by ELISA. Neutralizing activity of depleted sera was tested by FlowNT, as described above.

### ELISpot assay

Memory B cell ELISpots were performed using the Human IgG ELISpot Basic kit (catalog no. 3850-2H, MabTech). Cryopreserved PBMC were thawed and placed into culture for 6 days with R848 plus IL-2 in R10 medium (RPMI 1640, 10% FBS, penicillin/streptomycin, l-glutamine) to stimulate the production of antibody by memory B cells. MAIPSWU10 plates were coated with PBS (negative control), anti-IgG antibody (positive control), live virus (DENV-1 strain WestPac74, DENV-2 S16803, DENV-3 CH53489, and DENV-4 TVP-360), or 15 µg/mL recombinant E (rE) protein from DENV-1–4 (Hawaii Biotech) and incubated overnight at 4 °C. After washing off the unbound virus, 100,000–200,000 harvested memory B cells were plated into each well of the plate. Plates were incubated at 37 °C overnight, after which cells were removed and spots were developed using horseradish peroxidase-conjugated anti-IgG secondary antibody and TMB substrate. Spots were counted using a CTL ImmunoSpot S6 Ultimate-V Analyzer (Cellular Technology Limited) and counts reported as spot-forming units per million input cells.

### Statistical analyses

Viremia curves were analyzed as log_10_-transformed values using a mixed-effects model with Geisser–Greenhouse correction and Dunnett’s multiple comparisons test. Peak viremia log_10_-transformed values were analyzed using Welch’s ANOVA model and Dunnett’s T3 multiple comparisons test. All viremia comparisons were made only against the control (mock primed) group, and individual variances were computed for each comparison. Fold-ADE values and log_10_-transformed neutralizing antibody values were compared across all groups using a one-way ANOVA model and Tukey’s multiple comparisons test with a single pooled variance. Spearman correlations were assessed for significance using a two-tailed *p* value. All tests for significance used an *α* = 0.05. Nonlinear regression analyses were performed using least squares regression without weighting; constraints were as described in the Section “Results”. All analyses utilized two-sided tests unless otherwise noted. All analyses were performed using GraphPad Prism 8.1.0 for Windows.

### Disclaimer

Material has been reviewed by the Walter Reed Army Institute of Research. There is no objection to its presentation and/or publication. The opinions or assertions contained herein are the private views of the author, and are not to be construed as official, or as reflecting true views of the Department of the Army or the Department of Defense.

### Reporting summary

Further information on research design is available in the Nature Research Reporting Summary linked to this article.

## Supplementary information

Supplementary Information

Reporting Summary

## Data Availability

The datasets generated and/or analyzed during the current study are available from the corresponding author on reasonable request.

## References

[1] Gubler DJ (1998). Dengue and dengue hemorrhagic fever. Clin. Microbiol. Rev..

[2] Ho TS, Wang SM, Lin YS, Liu CC (2013). Clinical and laboratory predictive markers for acute dengue infection. J. Biomed. Sci..

[3] Lei HY (2001). Immunopathogenesis of dengue virus infection. J. Biomed. Sci..

[4] Rashmi MV, Hamsaveena (2015). Haematological and biochemical markers as predictors of dengue infection. Malays. J. Pathol..

[5] Wilder-Smith A, Earnest A, Paton NI (2004). Use of simple laboratory features to distinguish the early stage of severe acute respiratory syndrome from dengue fever. Clin. Infect. Dis..

[6] Stanaway JD (2016). The global burden of dengue: an analysis from the Global Burden of Disease Study 2013. Lancet Infect. Dis..

[7] Guzman MG, Harris E (2015). Dengue. Lancet.

[8] Vannice KS, Durbin A, Hombach J (2016). Status of vaccine research and development of vaccines for dengue. Vaccine.

[9] Gailhardou S (2016). Safety overview of a recombinant live-attenuated tetravalent dengue vaccine: pooled analysis of data from 18 clinical trials. PLoS Negl. Trop. Dis..

[10] Lopez P (2016). Immunogenicity and safety of yellow fever vaccine (Stamaril) when administered concomitantly with a tetravalent dengue vaccine candidate in healthy toddlers at 12-13 months of age in colombia and peru: a randomized trial. Pediatr. Infect. Dis. J..

[11] Olivera-Botello G (2016). Tetravalent dengue vaccine reduces symptomatic and asymptomatic dengue virus infections in healthy children and adolescents aged 2-16 years in Asia and Latin America. J. Infect. Dis..

[12] Whitehead SS (2016). Development of TV003/TV005, a single dose, highly immunogenic live attenuated dengue vaccine; what makes this vaccine different from the Sanofi-Pasteur CYD vaccine?. Expert Rev. Vaccines.

[13] Durbin AP (2016). A 12-month-interval dosing study in adults indicates that a single dose of the national institute of allergy and infectious diseases tetravalent dengue vaccine induces a robust neutralizing antibody response. J. Infect. Dis..

[14] Kirkpatrick BD (2016). The live attenuated dengue vaccine TV003 elicits complete protection against dengue in a human challenge model. Sci. Transl. Med..

[15] Kirkpatrick BD (2015). Robust and balanced immune responses to all 4 dengue virus serotypes following administration of a single dose of a live attenuated tetravalent dengue vaccine to healthy, flavivirus-naive adults. J. Infect. Dis..

[16] Durbin AP (2013). A single dose of any of four different live attenuated tetravalent dengue vaccines is safe and immunogenic in flavivirus-naive adults: a randomized, double-blind clinical trial. J. Infect. Dis..

[17] Sirivichayakul C (2016). Safety and Immunogenicity of a tetravalent dengue vaccine candidate in healthy children and adults in dengue-endemic regions: a randomized, placebo-controlled phase 2 study. J. Infect. Dis..

[18] George SL (2015). Safety and Immunogenicity of a live attenuated tetravalent dengue vaccine candidate in flavivirus-naive adults: a randomized, double-blinded phase 1 clinical trial. J. Infect. Dis..

[19] Osorio JE, Partidos CD, Wallace D, Stinchcomb DT (2015). Development of a recombinant, chimeric tetravalent dengue vaccine candidate. Vaccine.

[20] Rupp R (2015). Safety and immunogenicity of different doses and schedules of a live attenuated tetravalent dengue vaccine (TDV) in healthy adults: a phase 1b randomized study. Vaccine.

[21] Capeding MR (2014). Clinical efficacy and safety of a novel tetravalent dengue vaccine in healthy children in Asia: a phase 3, randomised, observer-masked, placebo-controlled trial. Lancet.

[22] Sridhar S (2018). Effect of dengue serostatus on dengue vaccine safety and efficacy. N. Engl. J. Med..

[23] Villar L (2015). Efficacy of a tetravalent dengue vaccine in children in Latin America. N. Engl. J. Med..

[24] Normile D (2017). Safety concerns derail dengue vaccination program. Science.

[25] Iacobucci G (2018). WHO recommends additional tests for Sanofi’s dengue vaccine after safety concerns. Br. Med. J..

[26] Thomas SJ, Rothman AL (2015). Trials and tribulations on the path to developing a dengue vaccine. Vaccine.

[27] Putnak R (1996). Development of a purified, inactivated, dengue-2 virus vaccine prototype in Vero cells: immunogenicity and protection in mice and rhesus monkeys. J. Infect. Dis..

[28] Fernandez S (2015). An adjuvanted, tetravalent dengue virus purified inactivated vaccine candidate induces long-lasting and protective antibody responses against dengue challenge in rhesus macaques. Am. J. Trop. Med. Hyg..

[29] Lim SK, Lee YS, Namkung S, Lim JK, Yoon IK (2016). Prospects for dengue vaccines for travelers. Clin. Exp. Vaccin. Res..

[30] Martinez LJ (2015). Safety and immunogenicity of a dengue virus serotype-1 purified-inactivated vaccine: results of a phase 1 clinical trial. Am. J. Trop. Med. Hyg..

[31] Innis BL (1988). Virulence of a live dengue virus vaccine candidate: a possible new marker of dengue virus attenuation. J. Infect. Dis..

[32] Edelman R (1994). A live attenuated dengue-1 vaccine candidate (45AZ5) passaged in primary dog kidney cell culture is attenuated and immunogenic for humans. J. Infect. Dis..

[33] McKee KT (1987). Lack of attenuation of a candidate dengue 1 vaccine (45AZ5) in human volunteers. Am. J. Trop. Med. Hyg..

[34] Hoke CH (1990). Preparation of an attenuated dengue 4 (341750 Carib) virus vaccine. II. Safety and immunogenicity in humans. Am. J. Trop. Med. Hyg..

[35] Marchette NJ (1990). Preparation of an attenuated dengue 4 (341750 Carib) virus vaccine. I. Pre-clinical studies. Am. J. Trop. Med. Hyg..

[36] Edelman R (2003). Phase I trial of 16 formulations of a tetravalent live-attenuated dengue vaccine. Am. J. Trop. Med. Hyg..

[37] Kanesa-Thasan N (2003). Phase 1 studies of Walter Reed Army Institute of Research candidate attenuated dengue vaccines: selection of safe and immunogenic monovalent vaccines. Am. J. Trop. Med. Hyg..

[38] Sun W (2003). Vaccination of human volunteers with monovalent and tetravalent live-attenuated dengue vaccine candidates. Am. J. Trop. Med. Hyg..

[39] Sun W (2009). Phase 2 clinical trial of three formulations of tetravalent live-attenuated dengue vaccine in flavivirus-naive adults. Hum. Vaccines.

[40] Watanaveeradej V (2011). Safety and immunogenicity of a tetravalent live-attenuated dengue vaccine in flavivirus-naive infants. Am. J. Trop. Med. Hyg..

[41] Simasathien S (2008). Safety and immunogenicity of a tetravalent live-attenuated dengue vaccine in flavivirus naive children. Am. J. Trop. Med. Hyg..

[42] Thomas SJ (2013). A phase II, randomized, safety and immunogenicity study of a re-derived, live-attenuated dengue virus vaccine in healthy adults. Am. J. Trop. Med. Hyg..

[43] Watanaveeradej V (2014). Safety and immunogenicity of a rederived, live-attenuated dengue virus vaccine in healthy adults living in Thailand: a randomized trial. Am. J. Trop. Med. Hyg..

[44] Lin L (2020). Immunogenicity of a live-attenuated dengue vaccine using a heterologous prime-boost strategy in a phase I randomized clinical trial. J. Infect. Dis..

[45] Grunst MW (2020). Functional interactions of common allotypes of rhesus macaque FcgammaR2A and FcgammaR3A with human and macaque IgG subclasses. J. Immunol..

[46] Gromowski GD (2018). Delineating the serotype-specific neutralizing antibody response to a live attenuated tetravalent dengue vaccine. Vaccine.

[47] Eckels KH (2003). Modification of dengue virus strains by passage in primary dog kidney cells: preparation of candidate vaccines and immunization of monkeys. Am. J. Trop. Med. Hyg..

[48] Borges MB (2019). Detection of post-vaccination enhanced dengue virus infection in macaques: an improved model for early assessment of dengue vaccines. PLoS Pathog..

[49] Halstead SB, Shotwell H, Casals J (1973). Studies on the pathogenesis of dengue infection in monkeys. II. Clinical laboratory responses to heterologous infection. J. Infect. Dis..

[50] Halstead SB (1979). In vivo enhancement of dengue virus infection in rhesus monkeys by passively transferred antibody. J. Infect. Dis..

[51] Goncalvez AP, Engle RE, St Claire M, Purcell RH, Lai CJ (2007). Monoclonal antibody-mediated enhancement of dengue virus infection in vitro and in vivo and strategies for prevention. Proc. Natl. Acad. Sci. USA.

[52] Lobigs M (2010). An inactivated Vero cell-grown Japanese encephalitis vaccine formulated with Advax, a novel inulin-based adjuvant, induces protective neutralizing antibody against homologous and heterologous flaviviruses. J. Gen. Virol..

[53] Simmons M, Burgess T, Lynch J, Putnak R (2010). Protection against dengue virus by non-replicating and live attenuated vaccines used together in a prime boost vaccination strategy. Virology.

[54] Simmons M, Murphy GS, Kochel T, Raviprakash K, Hayes CG (2001). Characterization of antibody responses to combinations of a dengue-2 DNA and dengue-2 recombinant subunit vaccine. Am. J. Trop. Med Hyg..

[55] Simmons M, Porter KR, Hayes CG, Vaughn DW, Putnak R (2006). Characterization of antibody responses to combinations of a dengue virus type 2 DNA vaccine and two dengue virus type 2 protein vaccines in rhesus macaques. J. Virol..

[56] Valdes I (2019). A heterologous prime-boost strategy for immunization against Dengue virus combining the Tetra DIIIC subunit vaccine candidate with the TV005 live-attenuated tetravalent vaccine. J. Gen. Virol..

[57] Valdes I, Lazo L, Hermida L, Guillen G, Gil L (2019). Can complementary prime-boost immunization strategies be an alternative and promising vaccine approach against dengue virus?. Front. Immunol..

[58] Lu S (2009). Heterologous prime-boost vaccination. Curr. Opin. Immunol..

[59] Kardani K, Bolhassani A, Shahbazi S (2016). Prime-boost vaccine strategy against viral infections: mechanisms and benefits. Vaccine.

[60] Glass A (2020). The effects of Japanese encephalitis vaccine and accelerated dosing scheduling on the immunogenicity of the chimeric yellow fever derived tetravalent dengue vaccine: a phase ii, randomized, open-label, single-center trial in adults aged 18 to 45 years in the United States. J. Infect. Dis..

[61] Scott RM (1983). Dengue 2 vaccine: dose response in volunteers in relation to yellow fever immune status. J. Infect. Dis..

[62] Kayser M (1985). Human antibody response to immunization with 17D yellow fever and inactivated TBE vaccine. J. Med. Virol..

[63] Katzelnick LC, Montoya M, Gresh L, Balmaseda A, Harris E (2016). Neutralizing antibody titers against dengue virus correlate with protection from symptomatic infection in a longitudinal cohort. Proc. Natl. Acad. Sci. USA.

[64] Guzman MG (2007). Neutralizing antibodies after infection with dengue 1 virus. Emerg. Infect. Dis..

[65] Pletnev AG, Putnak R, Speicher J, Wagar EJ, Vaughn DW (2002). West Nile virus/dengue type 4 virus chimeras that are reduced in neurovirulence and peripheral virulence without loss of immunogenicity or protective efficacy. Proc. Natl. Acad. Sci. USA.

[66] McCracken MK (2017). Impact of prior flavivirus immunity on Zika virus infection in rhesus macaques. PLoS Pathog..

[67] Johnson AJ, Martin DA, Karabatsos N, Roehrig JT (2000). Detection of anti-arboviral immunoglobulin G by using a monoclonal antibody-based capture enzyme-linked immunosorbent assay. J. Clin. Microbiol..

[68] Priyamvada L (2016). Human antibody responses after dengue virus infection are highly cross-reactive to Zika virus. Proc. Natl. Acad. Sci. USA.

[69] Kraus AA, Messer W, Haymore LB, de Silva AM (2007). Comparison of plaque- and flow cytometry-based methods for measuring dengue virus neutralization. J. Clin. Microbiol..

[70] de Alwis R (2012). Identification of human neutralizing antibodies that bind to complex epitopes on dengue virions. Proc. Natl. Acad. Sci. USA.

[71] Klungthong C (2015). Monitoring and improving the sensitivity of dengue nested RT-PCR used in longitudinal surveillance in Thailand. J. Clin. Virol..

