# Supplementary material for: Enhanced dengue vaccine virus replication and neutralizing antibody responses in immune primed rhesus macaques
Source: NPJ Vaccines. 2021 May 21;6:77. doi: 10.1038/s41541-021-00339-y (PMC8140083; doi:10.1038/s41541-021-00339-y)
Supplement: Supplementary file 1 — Supplementary Information [file 41541_2021_339_MOESM1_ESM.pdf]

a

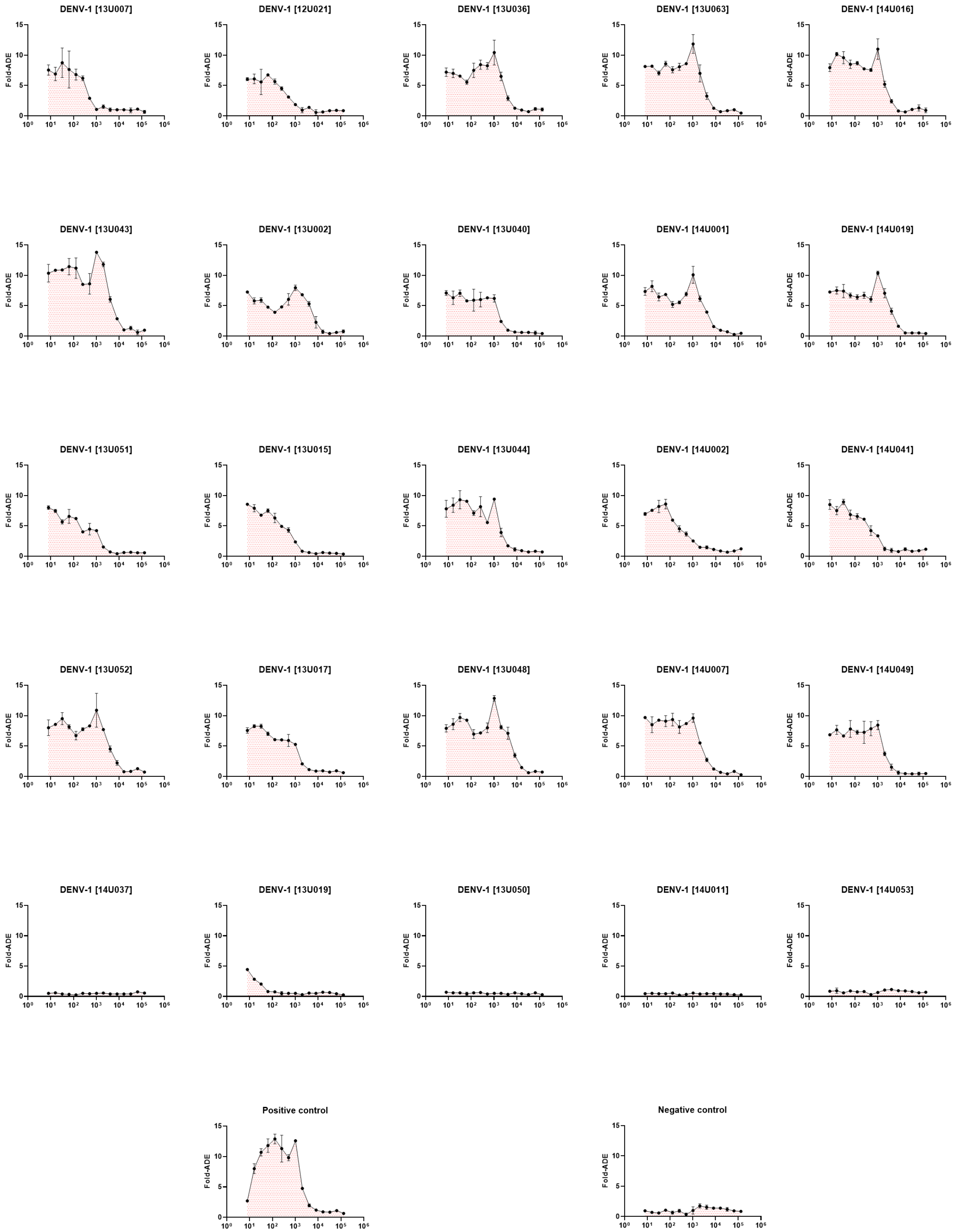
DENV-1 [13U007]
DENV-1 [12U021]
DENV-1 [13U036]
DENV-1 [13U063]
DENV-1 [14U016]
DENV-1 [13U043]
DENV-1 [13U002]
DENV-1 [13U040]
DENV-1 [14U001]
DENV-1 [14U019]
DENV-1 [13U051]
DENV-1 [13U015]
DENV-1 [13U044]
DENV-1 [14U002]
DENV-1 [14U041]
DENV-1 [13U052]
DENV-1 [13U017]
DENV-1 [13U048]
DENV-1 [14U007]
DENV-1 [14U049]
DENV-1 [14U037]
DENV-1 [13U019]
DENV-1 [13U050]
DENV-1 [14U011]
DENV-1 [14U053]
Positive control
Negative control
Fold-ADE
0
5
10
15
10^0
10^1
10^2
10^3
10^4
10^5
10^6

b

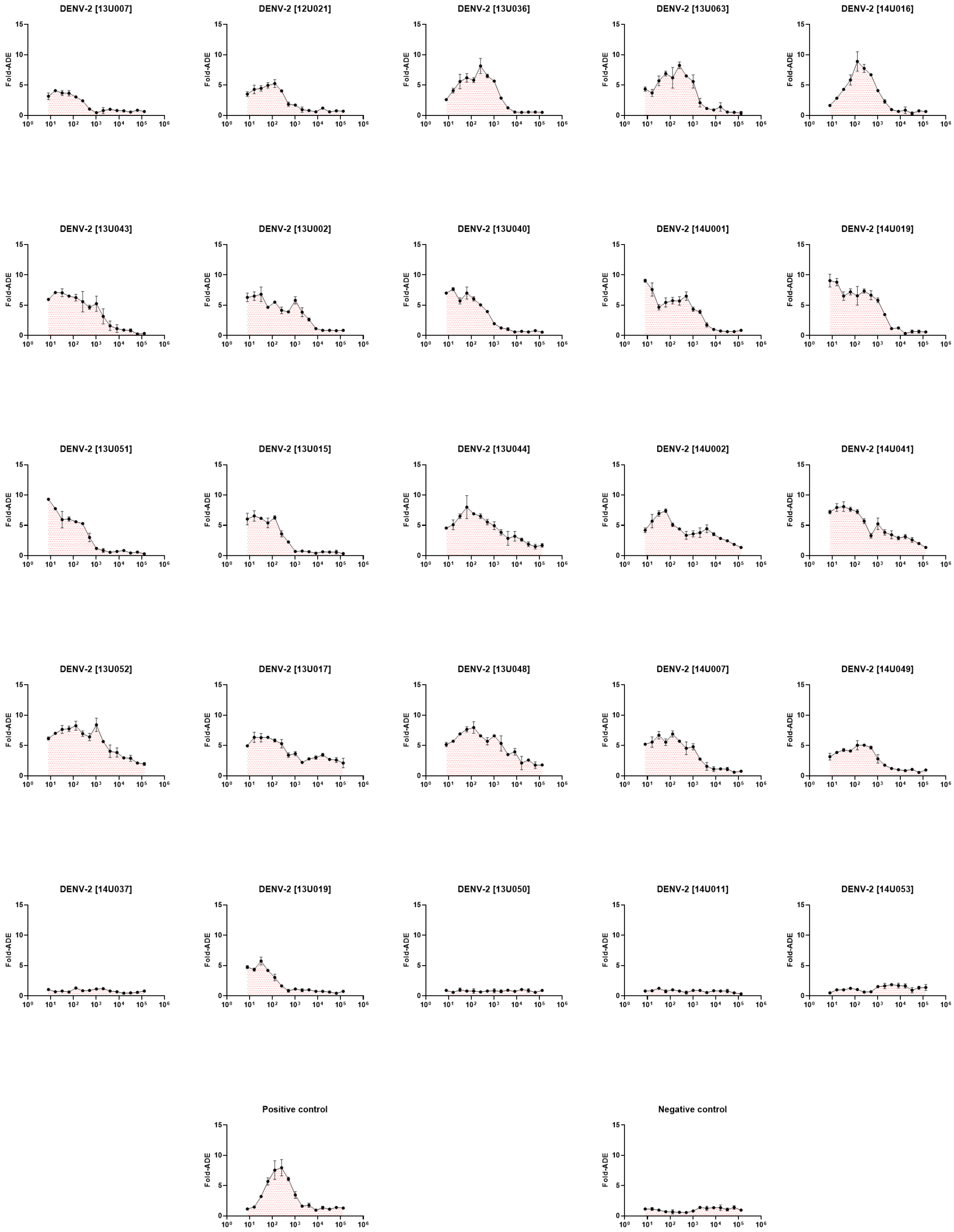
DENV-2 [13U007]
DENV-2 [12U021]
DENV-2 [13U036]
DENV-2 [13U063]
DENV-2 [14U016]
DENV-2 [13U043]
DENV-2 [13U002]
DENV-2 [13U040]
DENV-2 [14U001]
DENV-2 [14U019]
DENV-2 [13U051]
DENV-2 [13U015]
DENV-2 [13U044]
DENV-2 [14U002]
DENV-2 [14U041]
DENV-2 [13U052]
DENV-2 [13U017]
DENV-2 [13U048]
DENV-2 [14U007]
DENV-2 [14U049]
DENV-2 [14U037]
DENV-2 [13U019]
DENV-2 [13U050]
DENV-2 [14U011]
DENV-2 [14U053]
Positive control
Negative control
Fold-ADE
0
5
10
15
10^0 10^1 10^2 10^3 10^4 10^5 10^6

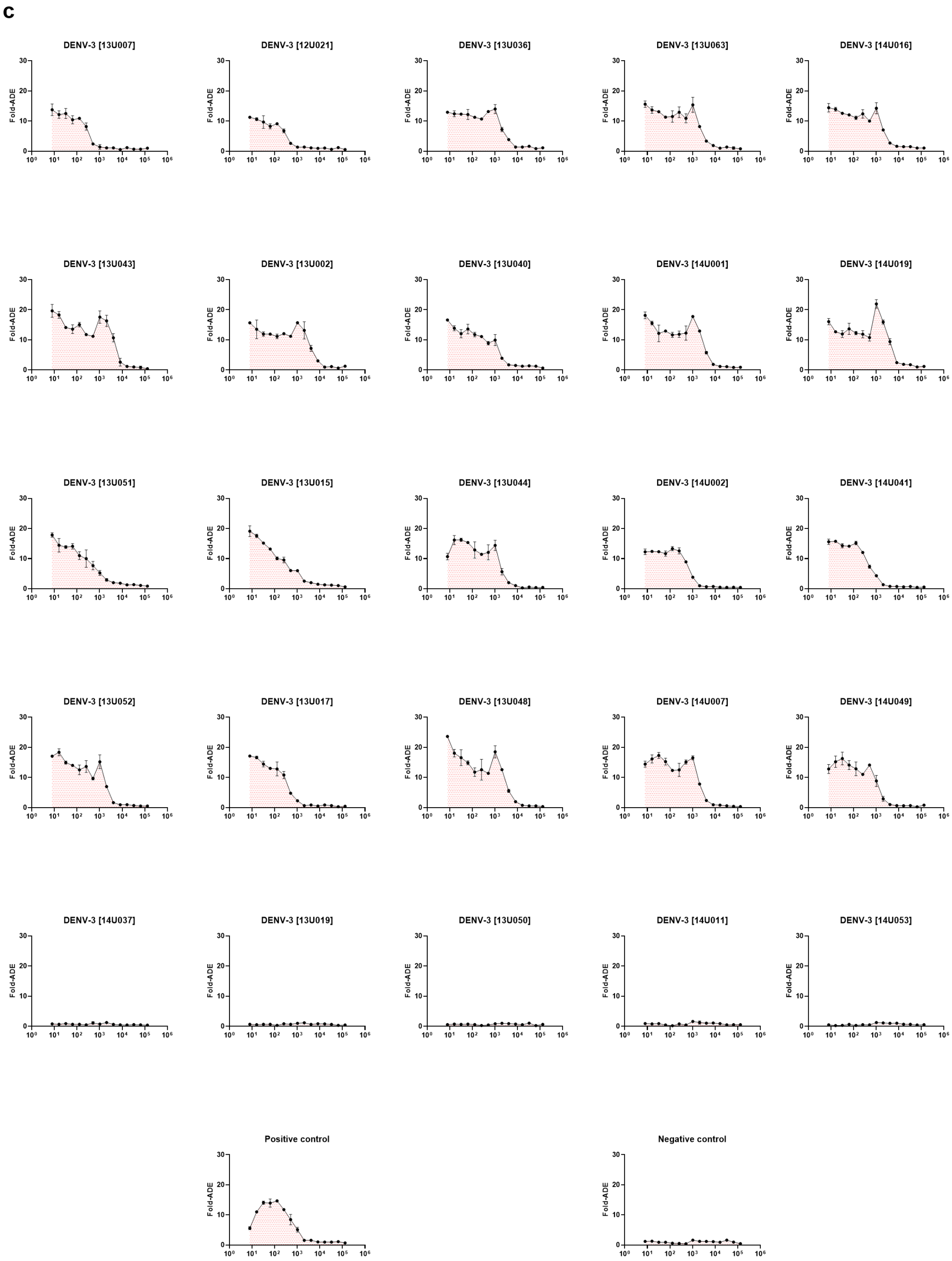
c
DENV-3 [13U007]
DENV-3 [12U021]
DENV-3 [13U036]
DENV-3 [13U063]
DENV-3 [14U016]
DENV-3 [13U043]
DENV-3 [13U002]
DENV-3 [13U040]
DENV-3 [14U001]
DENV-3 [14U019]
DENV-3 [13U051]
DENV-3 [13U015]
DENV-3 [13U044]
DENV-3 [14U002]
DENV-3 [14U041]
DENV-3 [13U052]
DENV-3 [13U017]
DENV-3 [13U048]
DENV-3 [14U007]
DENV-3 [14U049]
DENV-3 [14U037]
DENV-3 [13U019]
DENV-3 [13U050]
DENV-3 [14U011]
DENV-3 [14U053]
Positive control
Negative control
Fold-ADE
0
10
20
30
10^0 10^1 10^2 10^3 10^4 10^5 10^6

d

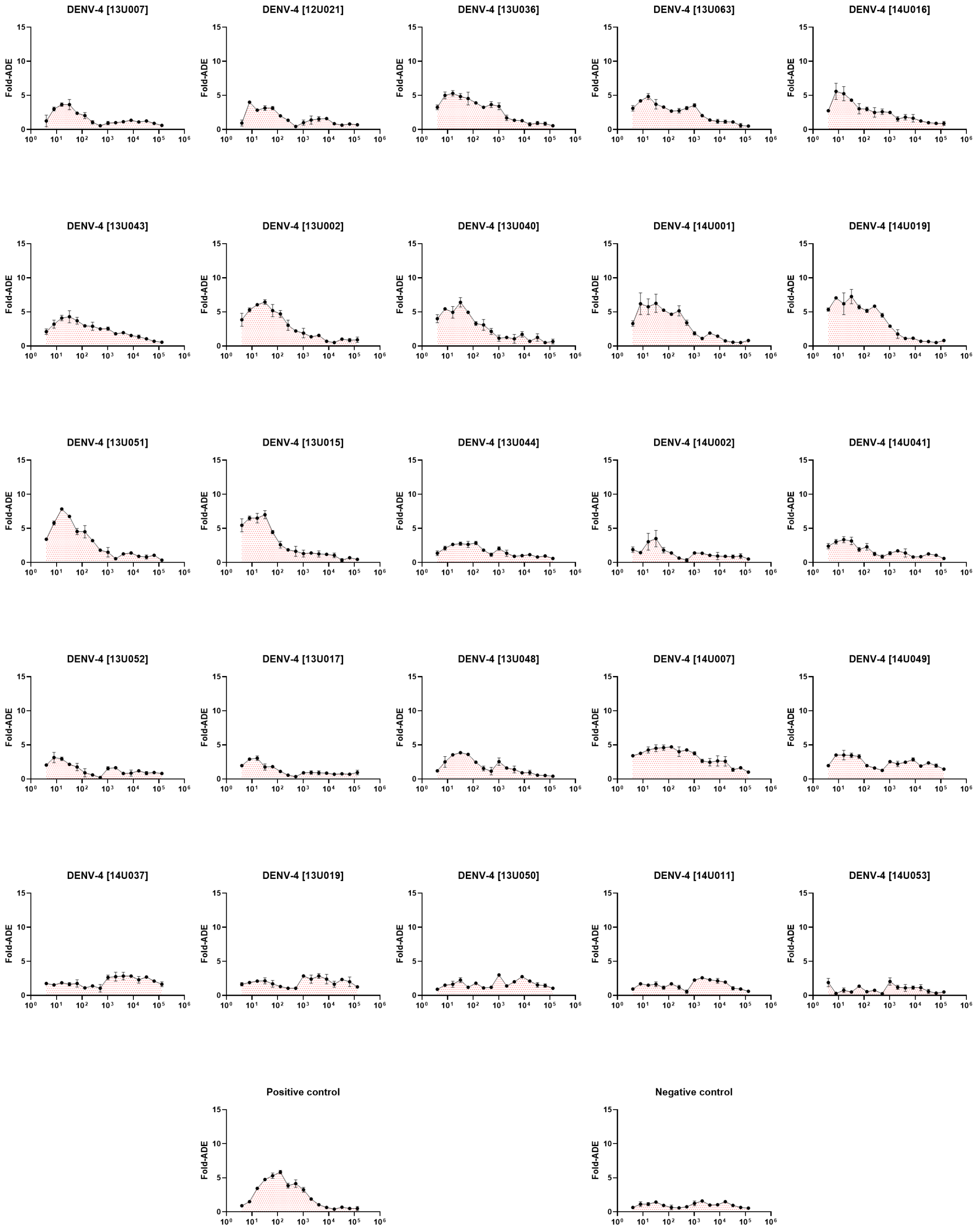
DENV-4 [13U007]
DENV-4 [12U021]
DENV-4 [13U036]
DENV-4 [13U063]
DENV-4 [14U016]
DENV-4 [13U043]
DENV-4 [13U002]
DENV-4 [13U040]
DENV-4 [14U001]
DENV-4 [14U019]
DENV-4 [13U051]
DENV-4 [13U015]
DENV-4 [13U044]
DENV-4 [14U002]
DENV-4 [14U041]
DENV-4 [13U052]
DENV-4 [13U017]
DENV-4 [13U048]
DENV-4 [14U007]
DENV-4 [14U049]
DENV-4 [14U037]
DENV-4 [13U019]
DENV-4 [13U050]
DENV-4 [14U011]
DENV-4 [14U053]
Positive control
Negative control
Fold-ADE
0
5
10
15
10^0
10^1
10^2
10^3
10^4
10^5
10^6

Supplementary Figure 1: In vitro enhancement of DENV infection by serum from individual animals. Full dilution curves from 1/8 to 1/131072 of sera collected on day 28 post-PIV vaccination are shown as fold-ADE against each of DENV-1-4 (panels **a**-**d**).

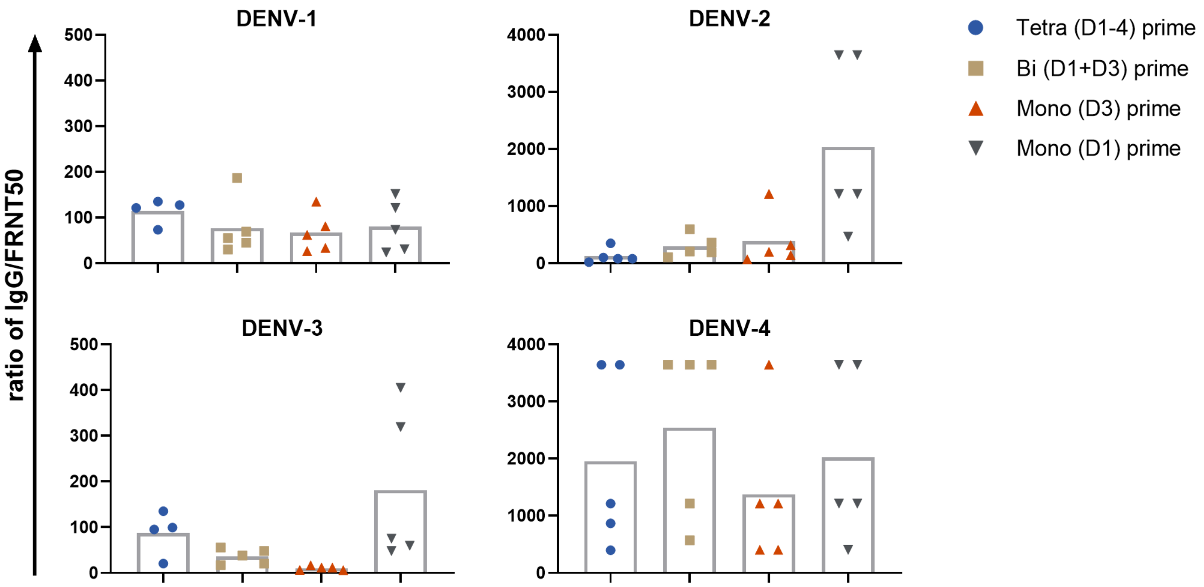

Supplementary Figure 2: Ratios of anti-DENV endpoint IgG titers to 50% neutralization titers of sera from day 28 post-PIV vaccination. Bar heights are the means for each group.

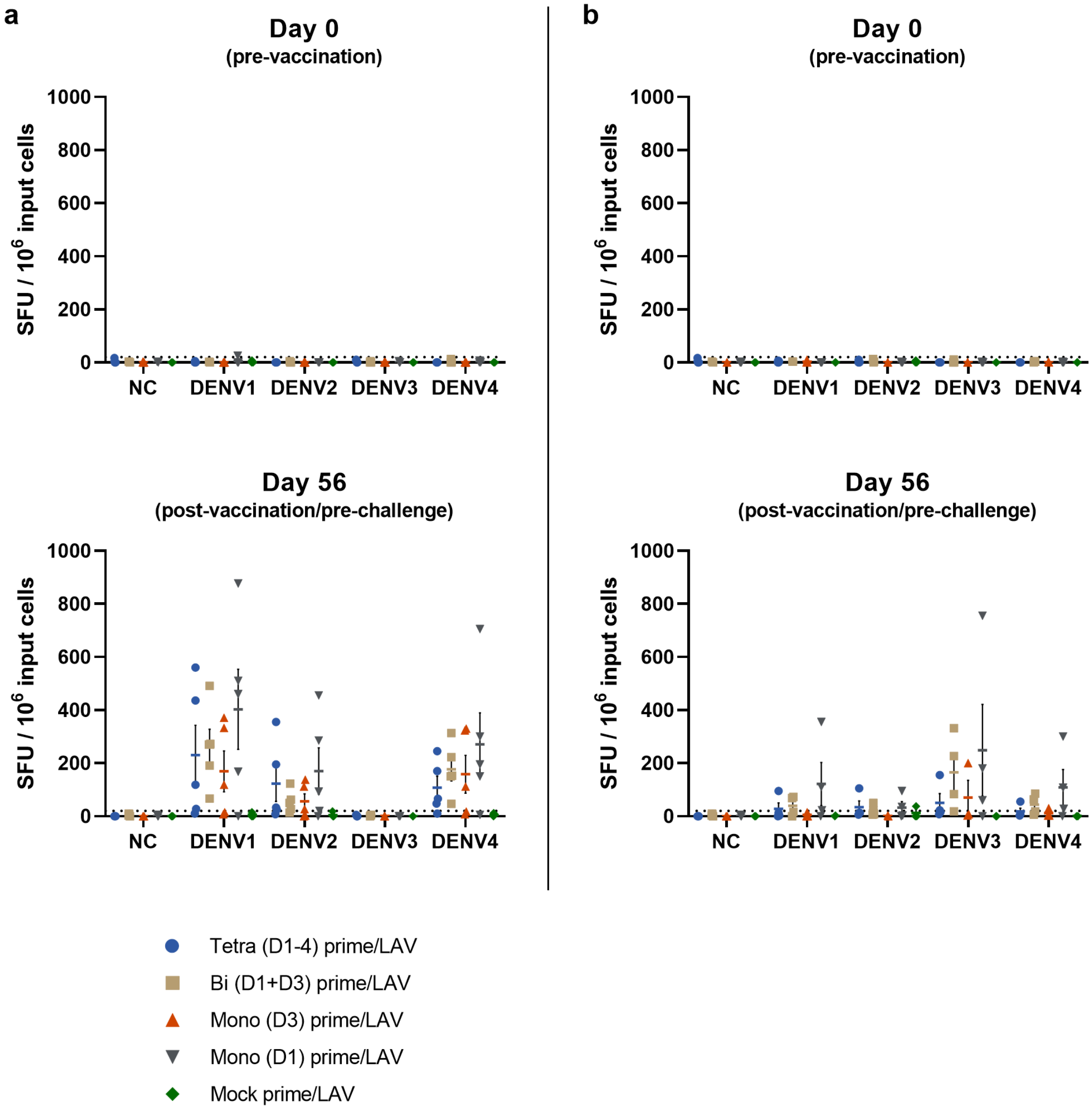

Supplementary Figure 3: Memory B cell response following LAV vaccination for individual DENV types. Blood samples collected on study days 0 and 56 (day 28 post LAV vaccination) were assessed for the concentration of memory B cells reacting to DENV-1-4 virions (**a**) or recombinant Envelope protein of DENV1-4 (**b**). Memory B cell concentrations are presented as spot-forming units (SFU)/10^6 input cells. Horizontal bars show the means of each group and associated error bars show standard error of the means.

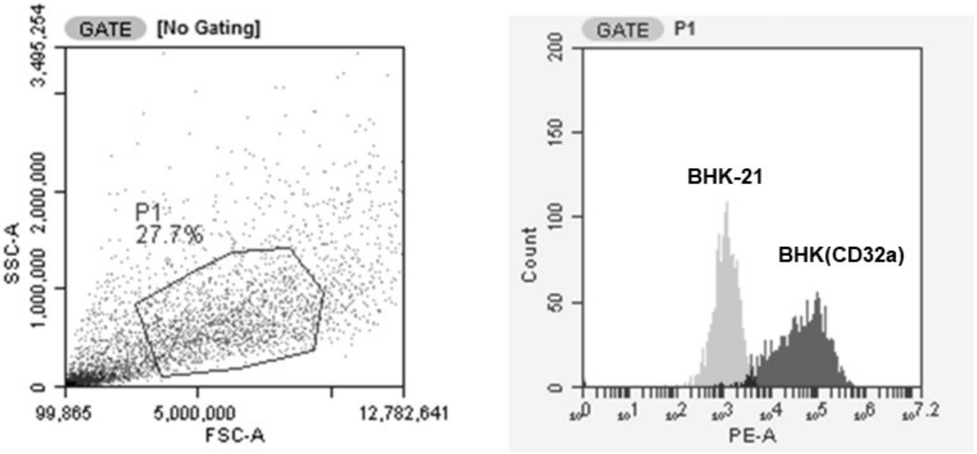

Supplementary Figure 4: Gating strategy used for monitoring BHK(CD32a) stable cell line.

CD32a-expressing cells were detected using a PE-conjugated anti-human CD32a mAb (Clone FLI8.26, BD Biosciences).

Supplementary Table 1: Geometric means of endpoint anti-DENV IgG titers from day 28 post-PIV vaccination.

Group	DENV-1	DENV-2	DENV-3	DENV-4
Tetra (D1-4) prime	1403	5852	1403	5852
Bi (D1+D3) prime	1951	5852	1951	5852
Mono (D3) prime	419	2430	419	1951
Mono (D1) prime	1566	4698	1566	3027

Supplementary Table 2: Confirmation of TDEN-LAV replication for each DENV type in both C6/36 and Vero cells. Shown are the titers on day 6 post-inoculation in genome equivalents (GE) per mL. All were undetectable on day 0.

	GE / mL	
Type	C6/36	Vero
DENV1	4.03E+08	1.14E+09
DENV2	3.46E+07	3.22E+09
DENV3	1.25E+09	1.21E+08
DENV4	3.56E+09	2.47E+10
