# Supplementary material for: Enhanced dengue vaccine virus replication and neutralizing antibody responses in immune primed rhesus macaques
Source: NPJ Vaccines. 2021 May 21;6:77. doi: 10.1038/s41541-021-00339-y (PMC8140083; doi:10.1038/s41541-021-00339-y)
Supplement: Supplementary file 2 — Reporting Summary [file 41541_2021_339_MOESM2_ESM.pdf]

nature research

Corresponding author(s): Gregory Gromowski

Last updated by author(s): Apr 2, 2021

Reporting Summary

Nature Research wishes to improve the reproducibility of the work that we publish. This form provides structure for consistency and transparency in reporting. For further information on Nature Research policies, see our Editorial Policies and the Editorial Policy Checklist.

Statistics

For all statistical analyses, confirm that the following items are present in the figure legend, table legend, main text, or Methods section.

n/a	Confirmed	
☐	☒	The exact sample size (*n*) for each experimental group/condition, given as a discrete number and unit of measurement
☐	☒	A statement on whether measurements were taken from distinct samples or whether the same sample was measured repeatedly
☐	☒	The statistical test(s) used AND whether they are one- or two-sided<br>*Only common tests should be described solely by name; describe more complex techniques in the Methods section.*
☒	☐	A description of all covariates tested
☐	☒	A description of any assumptions or corrections, such as tests of normality and adjustment for multiple comparisons
☐	☒	A full description of the statistical parameters including central tendency (e.g. means) or other basic estimates (e.g. regression coefficient) AND variation (e.g. standard deviation) or associated estimates of uncertainty (e.g. confidence intervals)
☒	☐	For null hypothesis testing, the test statistic (e.g. *F*, *t*, *r*) with confidence intervals, effect sizes, degrees of freedom and *P* value noted<br>*Give P values as exact values whenever suitable.*
☒	☐	For Bayesian analysis, information on the choice of priors and Markov chain Monte Carlo settings
☒	☐	For hierarchical and complex designs, identification of the appropriate level for tests and full reporting of outcomes
☒	☐	Estimates of effect sizes (e.g. Cohen's *d*, Pearson's *r*), indicating how they were calculated

Our web collection on statistics for biologists contains articles on many of the points above.

Software and code

Policy information about availability of computer code

Data collection	N/A
Data analysis	Data were analyzed using GraphPad Prism 8.1.0 for Windows as described in the Materials and Methods section.

For manuscripts utilizing custom algorithms or software that are central to the research but not yet described in published literature, software must be made available to editors and reviewers. We strongly encourage code deposition in a community repository (e.g. GitHub). See the Nature Research guidelines for submitting code & software for further information.

Data

Policy information about availability of data

All manuscripts must include a data availability statement. This statement should provide the following information, where applicable:

- Accession codes, unique identifiers, or web links for publicly available datasets
- A list of figures that have associated raw data
- A description of any restrictions on data availability

The datasets generated and/or analyzed during the current study are available from the corresponding author on reasonable request.

Field-specific reporting

Please select the one below that is the best fit for your research. If you are not sure, read the appropriate sections before making your selection.

☒ Life sciences ☐ Behavioural & social sciences ☐ Ecological, evolutionary & environmental sciences

For a reference copy of the document with all sections, see nature.com/documents/nr-reporting-summary-flat.pdf

Life sciences study design

All studies must disclose on these points even when the disclosure is negative.

Sample size	A power analysis was conducted based on a one-way ANOVA using historical data from prior rhesus macaque studies conducted at our facility.
Data exclusions	No data were excluded.
Replication	The power analysis was conducted to predetermine an adequate sample size to detect differences in NAb titers and viremia titers between groups. All in vitro assays were run in at least duplicate to ensure reproducibility of results.
Randomization	Animals were assigned in no particular order, while attempting to balance ages and weights, to the six experimental infection groups.
Blinding	Blinding was not necessary in this pre-clinical study.

Reporting for specific materials, systems and methods

We require information from authors about some types of materials, experimental systems and methods used in many studies. Here, indicate whether each material, system or method listed is relevant to your study. If you are not sure if a list item applies to your research, read the appropriate section before selecting a response.

Materials & experimental systems

n/a	Involved in the study	
☐	☒	Antibodies
☐	☒	Eukaryotic cell lines
☒	☐	Palaeontology and archaeology
☐	☒	Animals and other organisms
☒	☐	Human research participants
☒	☐	Clinical data
☒	☐	Dual use research of concern

Methods

n/a	Involved in the study	
☒	☐	ChIP-seq
☐	☒	Flow cytometry
☒	☐	MRI-based neuroimaging

Antibodies

Antibodies used	Antibody clone FLI8.26, PE-conjugated, from BD Biosciences (CatNo. 550586) was used to target CD32a. Polyclonal antibody A6029 from Sigma (CatNo. A6029) was used as the reporter antibody in ELISAs.
Validation	All relevant validation information and references is available on the manufacturers' websites at the following URLs.<br><br>https://www.bdbiosciences.com/us/applications/research/b-cell-research/surface-markers/human/pe-mouse-anti-human-cd32-fli826-also-known-as-826/p/550586<br><br>https://www.sigmaaldrich.com/catalog/product/sigma/a6029?lang=en&region=US

Eukaryotic cell lines

Policy information about cell lines

Cell line source(s)	ATCC
Authentication	Cell lines were authenticated by ATCC prior to purchase.
Mycoplasma contamination	Cell lines are tested mycoplasma negative by ATCC prior to purchase.
Commonly misidentified lines (See ICLAC register)	None.

Animals and other organisms

Policy information about studies involving animals; ARRIVE guidelines recommended for reporting animal research

Laboratory animals	Macaca mulatta, rhesus, female, <10 years of age
Wild animals	The study did not involve wild animals.
Field-collected samples	The study did not collect field samples.
Ethics oversight	The study was subject to WRAIR/NMRC Institutional Animal Care and Use Committee oversight.

Note that full information on the approval of the study protocol must also be provided in the manuscript.

Flow Cytometry

Plots

Confirm that:

- [x] The axis labels state the marker and fluorochrome used (e.g. CD4-FITC).
- [x] The axis scales are clearly visible. Include numbers along axes only for bottom left plot of group (a 'group' is an analysis of identical markers).
- [x] All plots are contour plots with outliers or pseudocolor plots.
- [x] A numerical value for number of cells or percentage (with statistics) is provided.

Methodology

Sample preparation	Outlined in Methods section
Instrument	BD Accuri C6 Plus
Software	The default BD Accuri C6 Plus software was used to collect data and generate percentages.
Cell population abundance	N/A
Gating strategy	See Supplementary Figure 4.

- [x] Tick this box to confirm that a figure exemplifying the gating strategy is provided in the Supplementary Information.
